# Examining the Threat of H5N1 Highly Pathogenic Avian Influenza to Human Health

**DOI:** 10.1016/j.chest.2025.10.030

**Published:** 2025-11-04

**Authors:** Juliette Blais-Savoie, Emily Halajian, Kuganya Nirmalarajah, Andra Banete, Juan C. Corredor, Jonathon D. Kotwa, Yaejin Lee, Sugandha Raj, Shayan Sharif, Nicole Mideo, Samira Mubareka

**Affiliations:** a Sunnybrook Research Institute, Toronto, ON, Canada; b Department of Laboratory Medicine and Pathobiology, University of Toronto, Toronto, ON, Canada; c Department of Pathobiology, University of Guelph, Guelph, ON, Canada; d Department of Ecology and Evolutionary Biology, University of Toronto, Toronto, ON, Canada

**Keywords:** acute respiratory infection, clade 2.3.4.4b, emerging pathogens, H5N1, H5Nx, One Health, viral zoonoses

## Abstract

**Topic Importance:**

The clade 2.3.4.4b highly pathogenic avian influenza (HPAI) virus H5N1 is the etiologic agent for an ongoing panzootic with a rapidly increasing number of human infections. Although morbidity and mortality in humans with this clade seems to be limited to date, previous HPAI H5N1 viruses have been associated with mortality rates of approximately 50% in humans. Not all cases of clade 2.3.4.4b influenza A(H5N1) HPAI in humans have been associated with known exposure to infected animals. Therefore, clinicians must be aware of the changing viral ecology, human risk factors, and clinical presentations associated with H5N1 viruses to facilitate early case recognition and management of clade 2.3.4.4b A(H5N1) HPAI infection in humans.

**Review Findings:**

Historic H5N1 presentations have involved multiorgan systemic disease, notably including severe neurological disease. Common symptoms associated with clade 2.3.4.4b A(H5N1) HPAI include conjunctivitis, fever, and upper respiratory tract infection. Exposure to infected dairy cattle is a novel risk factor.

**Summary:**

The rapid global spread of clade 2.3.4.4b A(H5N1) viruses has been associated with severe disease and high mortality in many farmed animal species and wildlife. The composite picture of emerging risk to human health comprises an unprecedented number of mammalian infections, viral adaptations to mammalian hosts, severe neuroinvasive disease in naturally infected mammals, and spillover into novel species such as dairy cows with forward transmission to humans. Preparedness measures are crucial to mitigating significant human health impacts from this virus and must include a Canadian One Health Training Program in Emerging Zoonoses approach that promotes both animal and human health.

Avian influenza viruses (AIVs) are influenza A viruses in the family *Orthomyxoviridae* that circulate primarily among avian hosts. Influenza A viruses are enveloped viruses with segmented negative-sense single-stranded RNA genomes consisting of 8 genomic segments, each encoding ≥ 1 viral proteins. The surface proteins of influenza A viruses include 18 known hemagglutinin and 11 known neuraminidase types. Hemagglutinin subtypes 1 through 16 and neuraminidase subtypes 1 through 9 are present in avian influenza viruses, and H17-18 and N10-11 are present in bat influenza viruses.^1^^,^^2^ Subtypes A(H1N1) and A(H3N2) circulate seasonally in humans.^3^ Lineage and clade classifications are based on the hemagglutinin, independently of other viral gene segments. Lineages are denoted by geographic location and host species. Clades, which exist within lineages, are assigned numerically and represent genetic differences of > 1.5%. Additional branches are added when a single clade evolves into multiple distinct subclades (eg, 2.3.4.4a and 2.3.4.4b are distinct subclades of clade 2.3.4.4).^4^

Viral entry into cells is primarily determined by interactions between the receptor binding site of the hemagglutinin and host cell sialic acid receptors—α2,3-linked galactose and α2,6-linked galactose—although this paradigm of preferential virus-host cell receptor interactions is being questioned as new putative receptors are discovered.^5^ The specificity of viral binding to these different receptors partially determines the host range of the virus. Conventionally, AIVs preferentially bind α2,3-linked galactose receptors and mammalian influenza viruses preferentially bind α2,6-linked galactose receptors.^6^ Exposure of the hemagglutinin fusion peptide for host cell entry requires cleavage of the hemagglutinin protein by host proteases. Low-pathogenic avian influenza (LPAI) viruses require trypsin-like proteases for cell entry, and therefore are confined to host tissues that express these proteases; in avian species, these are limited largely to respiratory and gastrointestinal epithelial cells. These viruses are associated with subclinical disease in both domestic and wild birds, enabling cryptic spread of LPAI. Conversely, highly pathogenic avian influenza (HPAI) viruses have acquired mutations associated with increased hemagglutinin cleavability by non-trypsin-like proteases that are distributed more ubiquitously, such as furin.^7^ This allows the virus to disseminate and cause systemic infection.

HPAI infections can cause a range of illness in wild birds, from subclinical to severe disease, and are associated with high mortality rates in domestic chickens. AIV subtypes H5 and H7 are predisposed to acquiring a polybasic cleavage site and becoming highly pathogenic, and thus are of greater concern.^8^ The reservoir species for AIVs are wild waterfowl, particularly Anseriformes (ducks and geese) and Charadriiformes (gulls and shorebirds), with the highest prevalence (up to 60%) frequently reported in dabbling ducks (*Anas* species).^9^

Influenza viruses have been responsible for numerous global pandemics because of their ability to adapt to new hosts, enabled by the reassortment of viral genome segments when host cells are coinfected by influenza viruses of different antigenic subtypes (antigenic shift). Since the start of the 20th century, 5 influenza pandemics have been described: 1918 (H1N1), 1957 (H2N2), 1968 (H3N2), 1977 (H1N1), and 2009 (H1N1).^10^^,^^11^ Pandemics are thought to emerge after reassortment among avian and mammalian influenza viruses. The most recent 2009 H1N1 pandemic virus likely originated after reassortment among classical swine H1N1, European avian-like swine H1N1, and triple-reassortant swine H1N2 viruses.^10^

## Literature Search

The articles cited in this review were sourced primarily through PubMed database searches using the terms *highly pathogenic avian influenza*, *HPAI*, *goose/guangdong*, *2.3.4.4b*, *H5*, and *H5N1*. Surveillance articles were limited to the current epizootic beginning in 2021. Background information and clinical articles were not constrained by time of publication. Primary research articles were preferred, with seminal articles selected where relevant. Information on official guidelines was sourced from governing body websites. Preprint articles and news announcements from official sources were used only for novel developments for which peer-reviewed publications were not yet available.

## Evidence Review

### Animal Models of Clade 2.3.4.4b Infection

Experimental approaches often are used to infer risk to human health posed by emerging influenza A viruses. Isolates of the contemporary clade 2.3.4.4b A(H5N1) virus from Canadian wildlife were found to replicate successfully in primary human airway epithelial cells. Additionally, these isolates were shown to cause lethal disease in experimental mice, deer mice, and ferrets, and 1 isolate, A/Red Tailed Hawk/ON/FAV-0473-4/2022, successfully transmitted between ferrets (D. Kobasa et al, unpublished data, 2023). Ferret model pathotyping of clade 2.3.4.4b A(H5N1) isolates from the southeastern US revealed a strong correlation between the number of North American gene segments present and pathogenicity and systemic viral spread in ferrets.^12^ Indeed, ferrets infected with highly virulent North American and Eurasian reassortant viruses showed the most severe and distributed central nervous system histopathologic characteristics, whereas replication of viruses retaining wholly Eurasian genomes seemed to be limited to the olfactory neuroepithelium.^12^ Ferret pathotyping with clade 2.3.4.4b A(H5N5) also showed systemic viral spread.^13^ Certain mutations of the *PB2* gene (*E627K*, *E627V*, and *D701N*) have been associated with severe disease in mammals.^14^ Given that swine are theorized to act as so-called mixing vessels for influenza because they are susceptible to both avian and human influenza viruses, swine are a species of special concern for the possible emergence of new reassortant strains of influenza A virus. A recent study in swine showed that although no clinical signs were detected, clade 2.3.4.4b A(H5N1) isolates from wild mammals could be transmitted between domestic pigs.^15^ Recently, clade 2.3.4.4b A(H5N1) outbreaks were identified in nearly 1,000 dairy cattle herds across 17 US states.^16^ Clade 2.3.4.4b A(H5N1) replicates primarily in bovine mammary glands, with transmission assumed to occur primarily by milk secretions. Experimental mice were infected systemically with high viral titers after ingesting milk contaminated with clade 2.3.4.4b A(H5N1).^17^^,^^18^

### Epidemiology

**Figure 1:**
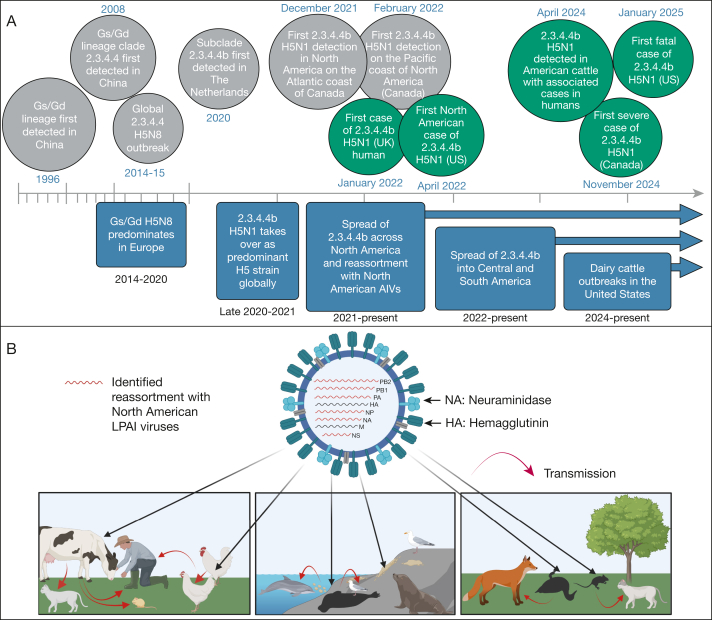
Clade 2.3.4.4b outbreak overview. A, Timeline of current A(H5Nx) panzootic. In 2020, a novel genotype, clade 2.3.4.4b A(H5N8), originally detected in Iraq, spread further into Europe, likely through bird migration.^23^ The evidence suggests that a clade 2.3.4.4b A(H5N8) influenza virus reassorted with Eurasian avian lineage LPAI viruses to give rise to the novel clade 2.3.4.4b A(H5N1), which was first detected in The Netherlands in late 2020. By the end of 2021, clade 2.3.4.4b A(H5N1) was the most predominant strain throughout Europe, Africa, and Asia. The first North American cases were detected in late 2021 and continued to spread to Central and South America in 2022 and 2023, then to Antarctica in late 2023.^13^^,^^24^ Global trends are shown in boxes below the timeline. First detections of viral spread in novel geographic regions or novel clades are shown in grey circles. Significant cases in humans are shown in teal circles. B, Diagram showing reassortment and viral ecology in the Americas. Since landing in North America, clade 2.3.4.4b A(H5) has reassorted with numerous North American LPAI viruses, acquiring gene segments (*PB2*, *PB1*, *PA*, *NP*, *NA*, and *NS*) from North American lineages.^12^^,^^14^^,^^25^ Most terrestrial mammals infected with clade 2.3.4.4b A(H5N1) have been observed in mesocarnivores (shown: fox, domestic cat), with consumption of infected bird carcasses such as waterfowl (shown), turkeys, or gulls seeming to be an important route of transmission. Widespread A(H5N1) infection of marine and coastal mammals also has been observed, which is suspected to have originated from fecal-oral transmission of clade 2.3.4.4b A(H5N1) through gull or shorebird guano or by direct contact at shared nesting and breeding grounds. Fecal runoff into coastal waters also is a possible route of transmission at highly populous breeding colonies, particularly on islands and islets. Transmission of clade 2.3.4.4b A(H5N1) from coastal mammals back to birds through direct contact also is possible. Positive findings in domestic cats have been observed, thus elevating the risk for pet owners and those occupationally exposed to infected animals.^26^ Transmission from agricultural animals to farm workers or other animals can occur through direct contact with infected animals. AIV = avian influenza virus; GsGd = A/Goose/Guangdong/1/1996; LPAI = low-pathogenic avian influenza.

The first human cases of A(H5N1) HPAI infection (A/Goose/Guangdong/1/1996 lineage) were reported in China in 1996 (Fig 1),^19^ with 18 cases and 6 deaths in humans.^20^ The *H5* genes since have diversified into clades 0 to 9 and have categorized further into subclades. Clade 2.3.4.4 A(H5Nx) subclades a through h have been responsible for epizootics (disease outbreaks in animals) from 2014 onward. Clade 2.3.4.4b has been under scrutiny given its association with high mortality rates among wild birds and poultry and extensive and sustained global spread in those species.^21^ Over time, 2.3.4.4b H5Nx viruses have reassorted with LPAI viruses, giving rise to multiple novel H5Nx genotypes.^22^

From January 2003 to December 2019, before the clade 2.3.4.4b A(H5N1) outbreak, 861 cases of human infection with A(H5N1) viruses (all clades) were reported with a case fatality rate of 53%.^27^ Although the rates of subclinical or otherwise undiagnosed infections are unknown, studies have found mean seroprevalence of 0% in the general population and up to 1.8% in populations exposed to infected poultry or other infected humans in Asia, Africa, and the Middle East between 1997 and 2020.^28^ The current risk assessment is that avian influenza poses a low risk to the general public.^29^

An outbreak of clade 2.3.2.1e A(H5N1) began in Cambodia in January 2025 involving 15 patients, 47% of whom died.^30^^,^^31^ One additional patient with clade 2.3.2.1e A(H5N1) was detected in Vietnam in April.^32^ These patients ranged in age from 3 to 65 years, and all were associated with prior exposure to domestic birds or domestic bird environments. Reported signs and symptoms include shortness of breath, fever, cough, and fatigue. The remainder of this review is focused on the ongoing outbreak of clade 2.3.4.4b A(H5N1), which, it should be noted, does not share the same vaccine and antiviral recommendations as clade 2.3.2.1e A(H5N1).^33^

From 2020 to 2022, 35 cases of clade 2.3.4.4b A(H5N8), A(H5N6), or A(H5N1) in humans were known, one of which represented the first domestically acquired H5N1 case in a human in the United States.^34^, ^35^, ^36^, ^37^, ^38^ Since 2023, 71 patients with highly pathogenic avian influenza virus clade 2.3.4.4b A(H5N1) have been identified in the United States, most of whom have exhibited mild to moderate disease (e-Table 1).^39^ In 1 series of 46 patients, 93% demonstrated conjunctivitis, 49% demonstrated fever, and 36% demonstrated respiratory symptoms. Several patients were asymptomatic.^40^ Of the 71 total patients, 42 patients reported exposure to infected cattle, and 24 patients reported exposure to commercial poultry.^39^ Exposure to cattle or commercial poultry operations thus may be indicative of potential clade 2.3.4.4b A(H5N1) infection; however, lack of exposure cannot rule out potential infection, as observed in 5 patients with unknown exposure or exposure to other animals.^39^^,^^41^

The first severe case of clade 2.3.4.4b A(H5N1) reported in North America and the only case in a human in Canada to date was a 13-year-old girl from British Columbia with asthma and an elevated BMI. The patient initially sought treatment with bilateral conjunctivitis in both eyes and a fever, then demonstrated cough, vomiting, and diarrhea. This was complicated by pneumonia, respiratory failure, acute kidney injury, thrombocytopenia, and leukopenia. The patient received combination antiviral therapy with oseltamivir, amantadine, and baloxavir, as well as plasma exchange and extracorporeal membrane oxygenation.^41^ In January 2025, the first fatal case of clade 2.3.4.4b A(H5N1) in the United States was reported in a patient from Louisiana who was hospitalized in December 2024 with severe respiratory illness after exposure to backyard poultry and wild birds.^42^ The first fatal case of 2.3.4.4b H5N1 in Mexico occurred in March 2025. The patient was a child from Durango who was younger than 10 years with no underlying medical conditions. Symptom onset included fever, malaise, and vomiting, followed by respiratory failure requiring hospitalization. Unspecified antiviral treatment was initiated 1 week after symptom onset; however, the patient later died as a result of respiratory complications.^43^ All 3 patients were infected with the D1.1 lineage of clade 2.3.4.4b A(H5N1). The B3.13 genotype, in addition to D1.1, has been circulating in US dairy cattle and has caused sporadic human infections, although none of these cases have been severe.

### Pathophysiology

**Figure 2:**
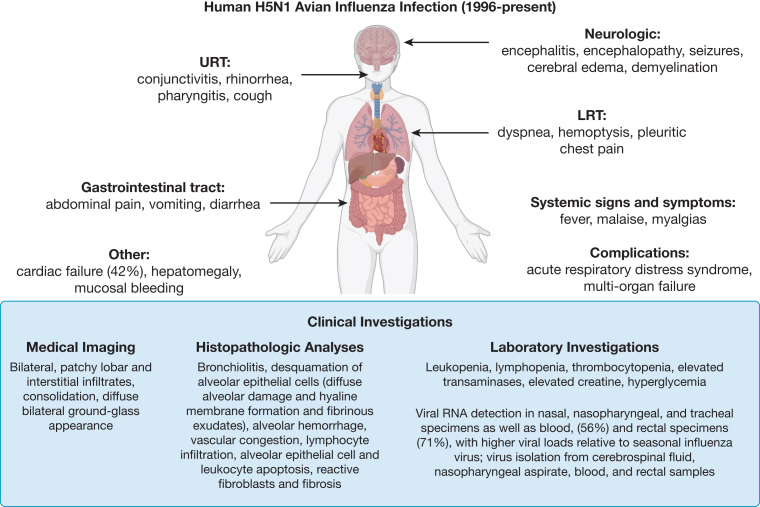
Diagram showing H5N1 infection in humans. Disease in humans ranges from asymptomatic to severe and may present with neurologic, respiratory, or gastrointestinal manifestations, or a combination thereof. The incubation period for H5N1 in humans is approximately 2 to 5 days, but can be up to 10 days. Initial signs and symptoms typically include nonspecific influenza-like illness, fever, and URT symptoms. In some patients, symptoms progress to more severe LRT disease. Progression may be secondary to cytokine dysregulation and pulmonary cellular infiltrates consisting predominantly of macrophages.^44^ Acute lung injury (ALI) is characterized by acute inflammation of the LRT followed by a fibroproliferative phase. ALI, when combined with pathogen-induced lung injury, may progress to ARDS.^45^ In addition to respiratory disease, A(H5N1) highly pathogenic avian influenza virus infection can involve extrapulmonary sites in humans, including neurologic symptoms such as cerebral edema and demyelination gastrointestinal symptoms such as diarrhea and lymphopenia.^44^^,^^45^ Human autopsies have revealed diffuse alveolar damage as well as pleural effusion, with gross lung weight of > 1,000 g on each side noted commonly.^47^ Viral dissemination beyond the respiratory tract to the CNS, lymphoid tissues, heart, liver, and spleen, as well as the placenta of 1 pregnant woman has been observed by pathologic analysis.^45^^,^^46^ Virus also could be detected in blood and rectal specimens. Viral load of URT specimens are significantly higher with more prolonged viral RNA shedding in patients with H5N1 when compared with patients with seasonal influenza.^44^ Timely identification and isolation of infected patients is essential and is enabled by a low threshold of suspicion for patients with consistent clinical presentations and epidemiologic factors, including exposure to sick birds or cattle within 10 days of symptom onset. Institutional or regional infection prevention and control should be consulted and followed. LRT = lower respiratory tract; URT = upper respiratory tract.

Clinical presentation and potential complications of clade 2.3.4.4b A(H5N1) infection, as well as pathogenesis (including viral replication kinetics and immunopathologic features), are outlined in Figure 2 and include data from infection with older clades.^20^^,^^44^, ^45^, ^46^, ^47^ Although typical presentations with influenza-like illness (fever, cough, sore throat) are not infrequent, atypical presentations have been described, including viral encephalitis, predominantly in pediatric patients.^47^ Gastrointestinal symptoms such as abdominal pain, vomiting, and diarrhea in the absence of respiratory symptoms before the onset of severe pneumonia also have been described.^45^^,^^47^ Findings on chest imaging have included focal consolidation, air bronchograms, bilateral patchy infiltrates, and opacities, as well as a ground-glass appearance in keeping with ARDS.^20^^,^^46^ In 1 early patient series, complications included elevated liver enzymes, coagulopathy, acute renal failure, and hemophagocytosis; lymphopenia was associated with poor outcomes in this study, as well as another prospective surveillance study from Thailand.^20^ Infection with bacterial pneumonia simultaneously or afterward does not seem to be a dominant feature based on historical data.^44^, ^45^, ^46^ Presentation with ocular symptoms has been noted commonly in recent patients, although not in historical case series.^48^, ^49^, ^50^

The α2,3-linked sialic acid receptor preference of AIVs plays a significant role in the pathogenesis and infection dynamics of AIV in humans. The human respiratory tract expresses primarily α2,6-linked receptors in the upper respiratory tract consisting of the nasal mucosa, paranasal sinuses, pharynx, trachea, and bronchi. α2,3-Linked sialic acid in humans can be found in the lower respiratory tract, including bronchiolar and alveolar cells, as well as neurons, the colonic epithelium, vascular and intestinal endothelial cells, and conjunctiva.^3^ Studies have shown H5N1 AIV have increased affinity to human pulmonary microvascular endothelial cells compared with H1N1 seasonal influenza virus.^51^

### Evaluation

Testing for H5N1 should be considered for patients with acute respiratory illness, conjunctivitis, or both with a relevant exposure history. For patients with conjunctivitis and acute respiratory illness, conjunctival, nasopharyngeal, and combination nasal and oropharyngeal swabs should be collected. For patients demonstrating only conjunctivitis, only conjunctival and nasopharyngeal swabs are recommended.^52^ Standard testing for influenza A virus includes real-time polymerase chain reaction (PCR) targeting the highly conserved viral matrix gene. To determine viral subtype, laboratories may use PCR targeting specific hemagglutinin proteins; H1-, H3-, H5-, and H7-specific primers are used commonly. The World Health Organization and Centers for Disease Control and Prevention (CDC) have published several PCR primer sets, including universal influenza A primers with a matrix protein target, and several H5 primers sets, including one designed for increased sensitivity to clade 2.3.4.4b A(H5N1) strains.^53^^,^^54^ However, individual diagnostic or surveillance laboratories often use either commercial or laboratory-developed tests, often optimized for regionally circulating viruses, sample type, and workflow. Nontypeable (matrix-positive, H1-negative, H3-negative) and H5-positive samples should be sent to public health laboratories and may be referred for whole genome sequencing for more fulsome identification, risk analysis, and genomic epidemiologic analysis.^55^ Such analyses have shown rapid evolution of H5N1 viruses in human hosts.^55^^,^^56^ Serologic assays are a critical tool for surveillance and vaccine trials, but are used rarely for individual patient diagnosis. They have been used to discern false-positive PCR results from environmental contamination.^57^ Virus isolation requires higher levels of biosafety and biosecurity, and thus is performed only in higher biosecurity laboratories according to jurisdictional regulations. Numerous influenza A virus rapid antigen tests are commercially available (e-Table 3); however, only one such test allows for H5 subtyping: the AVantage/H5N1 Flu Test (Arbor Vita Corporation).^58^ This test is designed to target the viral nonstructural-1 protein, which may lead to issues with sensitivity and specificity with reassortant viruses. Rapid antigen tests often are less sensitive than PCR testing, particularly with novel viruses, and the AVantage/H5N1 Flu Test was not designed for clade 2.3.4.4b A(H5N1) because it was approved before the outbreak.^58^ Diagnostic tests should be covered by most US insurers.^59^ Patients with positive findings may go undetected if they do not seek diagnosis or treatment in the absence of access to health care or because they experience only mild symptoms.

### Treatment Considerations

Antivirals are used to reduce symptom duration and to improve outcomes in at-risk patients with influenza and to mitigate the risk of transmission.^44^ Influenza antivirals also may be effective in preventing infection when used as prophylaxis before or after exposure.^60^ Treatment options for human H5N1 infections include neuraminidase inhibitors (oseltamivir, zanamivir, and peramivir) and baloxavir (cap-dependent endonuclease inhibitor).^60^, ^61^, ^62^

The CDC recommends oseltamivir for mild to severe disease at standard dosing, although a prolonged course may be considered for severe disease and patients with immunosuppression. On July 19, 2024, the CDC announced Emergency Use Instructions for H5N1 treatment and postexposure prophylaxis (PEP) with oseltamivir. Differences regarding the dosages and durations of oseltamivir for treatment vs PEP are notable. The Emergency Use Instructions recommend oseltamivir treatment initiation after 48 hours from symptom onset, although initiation beyond this window is still recommended, particularly for those with severe disease.^61^ Oseltamivir treatment for longer durations (eg, 10 days) is recommended for hospitalized patients based on clinical and virologic data.^61^ For PEP, a higher total daily dose and flexible duration with a dosing regimen of bid is recommended in patients who are asymptomatic with close contacts who have confirmed or probable infection or asymptomatic people who are exposed to H5N1-infected animals. PEP duration for 5 days is recommended if exposure was time limited and not continuous. For ongoing exposure (eg, household setting), 10 days is recommended.^61^

Although oseltamivir remains the recommended first-line treatment for severe H5N1 infections, various viral mutations are associated with oseltamivir resistance and reduced susceptibility. Historically, the *H274Y* mutation in the H5N1 neuraminidase was associated with high rates of oseltamivir resistance.^45^^,^^63^ In vitro work has revealed that H5N1 hemagglutinin clade 2 viruses have lower susceptibility to oseltamivir compared with clade 1 viruses.^64^ Known neuraminidase inhibitor resistance mutations in H5N1 include *E119A*, *H275Y*, and *N294S* mutations in neuraminidase. Human H5N1 virus isolates from Egypt with the N294S mutation exhibited a 57-fold to 138-fold decrease in oseltamivir susceptibility based on 3 neuraminidase inhibition assays.^65^ *H275Y* is another known mutation associated with oseltamivir resistance, whereas the *E119A* mutation has been linked to zanamivir resistance. H5N1 mutations associated with reduced susceptibility to neuraminidase inhibitors also include *I117V*, *K150N*, *I222V/T/K*, and *S246N*.^64^ The *I117V* mutation in H5N1 was found to reduce oseltamivir sensitivity by fivefold to 16-fold and to reduce zanamivir sensitivity up to fourfold.^66^ In vivo studies in mice suggest that the IV neuraminidase inhibitor peramivir has therapeutic efficacy against both H5N1 wildtype and virus with the neuraminidase *H275Y* mutation.^67^ Available sequence data indicate a low frequency of neuraminidase inhibitor resistance variants, with the exception of a D1.1 lineage H5N1 strain detected in British Columbia poultry that acquired the *H275Y* neuraminidase mutation through reassortment with North American LPAI viruses.^25^ Amantadine and other adamantane derivatives have been linked to increased occurrence of viral resistance and therefore are not recommended as monotherapy for H5N1 infection.^64^

Baloxavir is the most recently approved antiviral and exhibits broad activity against various strains and subtypes of influenza viruses. When compared with oseltamivir, treatment with baloxavir in H5N1-infected mice resulted in a significant decrease of viral titers in the brain, lungs, and kidneys, resulting in reduced mortality. In A(H5N1)-infected mice, oseltamivir and baloxavir combination therapy exerted a more potent effect on viral replication and improved survival in comparison with oseltamivir or baloxavir monotherapies. These findings suggest that baloxavir has potent antiviral efficacy and that combination therapy is effective in reducing viral replication. However, resistance has been shown to evolve rapidly with baloxavir use, posing a potential risk.^68^ In immunocompromised patients with prolonged H5N1 shedding and patients with severe disease resulting from A(H5N1), combination therapy with differing mechanisms of action may be beneficial. Other agents including inhaled zanamivir, IV peramivir, or oral baloxavir also may be considered.

### Preparedness and Countermeasures

A key line of defence against avian influenza spread is the rapid detection, reporting, and containment of cases and outbreaks in animals (e-Table 2). Additionally, public and animal health agencies globally are conducting genomic surveillance for reassortments and mutations associated with mammalian adaptation and antiviral resistance. Viral circulation in wildlife and livestock poses a continued risk for sporadic human infection. Risk factors for humans include handling sick poultry for cooking, holding or playing with unwell poultry or ducks, and consuming poultry products that are raw or undercooked. Exposure to infected dairy cattle or unpasteurized milk now is an additional consideration and underscores the importance of strongly supporting surveillance and mitigation measures.^60^

Numerous pandemic H5N1 vaccines have been developed for potential use in human populations in the event of an H5N1 outbreak. These include inactivated whole virion, inactivated split (partial) virion, inactivated subunit, inactivated surface antigen, and live-attenuated vaccines.^69^^,^^70^ The United States maintains a stockpile of vaccines against A(H5N1) and A(H7N9) viruses, whereas some countries opt to maintain negotiated agreements for access to vaccines from vaccine manufacturers in the event of a pandemic.^69^^,^^70^ The Sanofi Pasteur A(H5N1) vaccine, which has been licensed as the US national A(H5N1) pandemic stockpile vaccine, uses inactivated surface antigen from the A/Vietnam/1203/2004 strain.^71^ The United States also has approved the Arepanrix (ID Medical Corporation of Quebec) and Audenz (Seqirus) monovalent A(H5N1) vaccines.^72^ Single doses of A(H5) avian influenza vaccines seem less immunogenic in naive humans than seasonal influenza vaccines, requiring a second booster dose or use of an adjuvant for a robust immune response. Adjuvants MF59, ASO_3_, and Al(OH)_3_ have been shown to improve H5 vaccine response and are stockpiled for potential use in a pandemic. Use of adjuvants may reduce the necessary amount of antigen per dose, extending resources. Although the US government has not published a vaccine rollout strategy for the current outbreak, it stockpiles enough H5 antigen and adjuvants to immunize critical care workers and other at-risk populations rapidly.^69^^,^^73^, ^74^, ^75^ Finland began offering Seqirus H5 vaccines to at-risk poultry and fur farm workers in June 2024.^69^^,^^73^^,^^74^^,^^76^ Canada recently sourced Arepanrix H5N1 (A/American wigeon clade 2.3.4.4b) vaccines and updated their preliminary guidance and deployment of human vaccination against H5N1 in humans to prioritize laboratory workers handling clade 2.3.4.4b A(H5N1) and those with ongoing contact with infected animals.^77^

Vaccines for use against A(H5N1) in birds also have been developed, and the United States maintains a stockpile of these as well. However, the World Organization for Animal Health suggests that vaccination of livestock is not a sustainable solution to combat avian influenza. The concern is that overuse of vaccines to curb active outbreaks could impact case detection and surveillance. When vaccines reduce disease severity, but not infection, a buildup of undetected subclinical cases may become established, which would remain a concern for spillover to humans.^78^ The European Food and Safety Authority has outlined a number of vaccination strategies for domestic poultry.^79^

## Future Directions

At this time, the risk posed to the general public from AIV is considered low.^29^ However, that reality could change at any time given the rapidly evolving situation involving infection in mammals and substantial knowledge gaps surrounding clade 2.3.4.4b A(H5N1) and human infections.

The frequent presence of migratory gulls and waterfowl in human-altered landscapes secondary to wetland habitat loss may lead to increased contact with livestock and humans, increasing spillover risk.^9^ The myriad of possible transmission interfaces that exist between humans and animals, both wild and domestic, means that our success at combating avian influenza virus outbreaks is tied intrinsically to the health of other animals.

The virulence of the clade 2.3.4.4b A(H5N1) virus combined with the current spillover pressure necessitate efforts to prepare for sporadic and sustained viral activity in human populations. Although it is suggested that countries maintain their own stockpiles of avian influenza vaccines and antivirals, many countries lack the necessary capacity. The uneven global distribution of vaccine manufacturing capacity further contributes to these inequities, which have persisted after the COVID-19 pandemic. Both international and interspecies spread of clade 2.3.4.4b A(H5N1) virus have been key components of the current panzootic. This landscape necessitates cross-sectoral, multidisciplinary, and international collaboration for mitigation efforts to work as effectively as possible.

## Summary

The ongoing clade 2.3.4.4b A(H5N1) outbreak has exhibited several concerning features, namely, sustained and rapid global spread, high pathogenicity in some mammalian species, infection of novel mammalian host species including dairy cattle, and reports of antiviral resistance. Historically, influenza A(H5N1) presentation in humans has included severe respiratory disease with gastrointestinal and neurologic involvement. Cases associated with the current outbreak have largely been mild, with most patients demonstrating conjunctivitis and respiratory symptoms. Clinicians should be aware of the range of presentations associated with A(H5N1) infection in humans, as well as the risk posed by exposure to infected poultry or cattle and the possibility of infection in the absence of known exposure.

## Funding/Support

J. B.-S. is the recipient of a Canadian Institutes for Health Research (CIHR) Doctoral Research Award, a CIHR Canadian Graduate Scholarship-Masters award, an Institute for Pandemics Graduate Studentship, and a Canadian One Health Training Program in Emerging Zoonoses Scholarship. E. H. is the recipient of a Canadian One Health Training Program in Emerging Zoonoses Scholarship. K. N. is the recipient of an Institute for Pandemics Graduate Studentship award and an Emerging and Pandemic Infections Consortium Researcher Mobility Award. A. B. is the recipient of a CIHR Postdoctoral Fellowship. J. D. K. is the recipient of a CIHR Postdoctoral Fellowship. Y. L. is the recipient of an Emerging and Pandemic Infections Consortium Doctoral Award. S. R. is the recipient of an Arrell Food Institute Graduate Scholarship. S. S. is supported by grants from the 10.13039/501100015516Ontario Agri-Food Innovation Alliance, 10.13039/501100000038Natural Sciences and Engineering Research Council of Canada (NSERC), 10.13039/100015101Chicken Farmers of Saskatchewan, Egg Farmers of Canada, Canadian Poultry Research Council, and University of Guelph’s Food from Thought initiative. N. M. is supported by an NSERC Discovery Grant. S. M. is a CIHR-Public Health Agency of Canada Applied Public Health Chair in Pandemic and Health Emergency Prevention, Preparedness, Response and Recovery and is funded by the CIHR [Planning and Dissemination Grant 183380 and Project Grant 186217] and the NSERC Alliance Grant [Grant 598055-24].

## Financial/Nonfinancial Disclosures

None declared.

## Supplementary Data

e-Online Data

e-Online Data

e-Online Data
