# Supplementary material for: Examining the Threat of H5N1 Highly Pathogenic Avian Influenza to Human Health
Source: Chest. 2025 Nov 4;169(4):947–57. doi: 10.1016/j.chest.2025.10.030 (PMC13084735; doi:10.1016/j.chest.2025.10.030)
Supplement: e-Online Data [file mmc1.docx]

**Examining the threat of H5N1 highly pathogenic avian influenza to human health**

Authors: Juliette Blais-Savoie, BSc^1,2^, Emily Halajian, MSc^1,2^, Kuganya Nirmalarajah, BHSc^1,2^, Andra Banete, PhD^1^, Juan C. Corredor, PhD^1^, Jonathon D. Kotwa, PhD^1^, Yaejin Lee, BSc^1,2^, Sugandha Raj, PhD^3^, Shayan Sharif, PhD DVM^3^, Nicole Mideo PhD^4^, Samira Mubareka, MD^1,2^

**Supplemental Material (e-Table 1)**

| **Sex** | **Time period** | **Age** | **Testing** | **Symptoms** | **Outcome** | **Treatment** | **Exposure** | **Location** | **Genotype or virus** | **Other** | **Ref** |
| --- | --- | --- | --- | --- | --- | --- | --- | --- | --- | --- | --- |
| male | December 2021 to January 2022 | early 80's | positive nasal swab for H5; WGS from human clinical respiratory sample, virus almost identical to duck viruses | asymptomatic | Recovered | prophylactic dose of oseltamivir (75 mg once per day), once tests confirmed AIV oseltamivir (75 mg twice per day) for 10 days | kept duck flock at their residence, animals infected & subsequently culled | United Kingdom | A/muscovy duck/England/074477/2021 | one close contact (no PPE) received post-exposure antiviral prophylaxis (oseltamivir; 75 mg once per day for 10 days), no symptoms | ^1^ |
| male | April 2022 | >18 and <40 years old | virus detected on one nasal specimen | fatigue | Recovered | oseltamivir | culling infected poultry | Colorado, USA |  | unclear if actual infection or if due to environmental contamination in nasal passage | ^2^ |
| male | September 2022 | 19 | nasopharyngeal sample tested positive (PCR) | asymptomatic | Recovered | isolation | worker at a poultry farm with H5N1 outbreak | Guadalajara, Spain |  | may have been environmental contamination; all close contacts tested negative | ^3^ |
| male | October 2022 | 27 | positive nasopharyngeal sample (PCR) | asymptomatic | Recovered | isolation | worker at a poultry farm with H5N1 outbreak | Guadalajara, Spain |  | may have been environmental contamination; all close contacts tested negative | ^3^ |
| female | October 2022 | 4 | laboratory-confimred H5 AIV; PCR test of endotracheal fluid | severe symptoms; cough, fever, jaundice, renal and liver failure | Recovered | Treated in intensive care unit; unknown if antivirals used | Family raised backyard poultry, and one week prior to the patient developing symptoms some poultry were sick and died | Viet Nam, Phú Thọ province |  |  | ^4^ |
| female | September to October 2022 | 38 | laboratory-confirmed H5N1 | severe pneumonia | Died | Unknown | exposure to backyard poultry, slaughtered a sick chicken | Guangxi province, China |  | Environmental samples were positive | ^4^ |
| female | December 2022 to January 2023 | 9 | nasopharyngeal sample, positive for H5 via RT-PCR | severe symptoms; conjunctival pruritus, coryza, nausea, vomiting, constipation, septic shock, pneumonia | Recovered | antivirals and mechanical ventilation | one week before symptom onset, family had acquired poultry that died without known cause. Other cases of backyard poultry dying in the same community where the patient's family lived were also reported | Bolívar Province, Ecuador |  |  | ^5^ |
| female | January to February 2023 | 53 | PCR positive sputum and BALF samples for H5N1 | severe pneumonia | Recovered | hospitalized, treated for severe acute respiratory distress syndrome, intratracheal intubation and mechanical ventilation, administered Peramivir and nirmatrelvir-ritonavir (for COVID) | history of contact with domestic poultry/exposure to backyard poultry | Jiangsu province, China |  | Co-infection with SARS-CoV-2 | ^6^ |
| male | March 2023 | 53 | laboratory confirmed, bronchoalveolar sample confirmed positive for H5 and later 2.3.4.4b H5N1 via PCR | cough and sore throat, pneumonia, respiratory distress | Recovered | hospitalized, mechanical ventilation | likely environmental exposure; report of sick and dead sea lions and wild birds near the patient's home, along with birds and sea lions in the vicinity testing positive | Antofagasta Region, Chile | Contained PB2 D701N and PB2 Q591K mutations |  | ^7^ |
| unknown | May 2023 | unknown | positive, later negative | asymptomatic | Recovered |  | poultry worker at a farm infected with HPAI H5N1 | United Kingdom |  | unclear if actual infection or environmental contamination of nose/throat; worker wore no PPE | ^8^ |
| unknown | May 2023 | unknown | positive, later negative | asymptomatic | Recovered | oseltamivir | culling infected poultry | United Kingdom |  | unclear if actual infection or environmental contamination of nose/throat; wore PPE | ^8^ |
| unknown | June 2023 | unknown | first sample negative, later sample H5 positive | sore throat, myalgia | Recovered |  | poultry worker at a farm infected with HPAI H5N1 | United Kingdom |  | unclear if actual infection or environmental contamination of nose/throat | ^9^ |
| unknown | July 2023 | unknown | first sample H5 positive, self-sample 2 days later negative | asymptomatic | Recovered |  | poultry worker at a farm infected with HPAI H5N1 | United Kingdom |  | unclear if actual infection or environmental contamination of nose/throat | ^9^ |
| male | April 2024 | >18 | PCR positive for both conjunctival and respiratory samples | conjunctivitis | Recovered | oseltamivir and isolation | worker at a commercial dairy cattle farm with direct contact with dairy cattle infected with HPAI H5N1. | Texas, USA | Had PB2 E627K mutation | Case was not wearing respiratory or eye PPE but wore gloves | ^10^ |
| unknown | May 2024 | >18 | nasopharyngeal sample negative but conjunctival sample psoitive | conjunctivitis | Recovered | antiviral treatment | exposure to infected milk while not wearing complete PPE; dairy farm worker | Michigan, USA |  | Not wearing full PPE when working directly with infected cows | ^11^ |
| unknown | May 2024 | >18 | nasopharyngeal sample PCR positive for H5, oropharyngeal and conjunctival negative | eye discomfort with watery discharge, cough without fever | Recovered | antiviral treatment | dairy farm worker; direct contact with infected dairy cow while not wearing complete PPE | Michigan, USA |  | Not wearing full PPE when working directly with infected cows | ^11^ |
| male | July 2024 | >18 | nasopharyngeal swab PCR positive for H5 | eye redness and irritation | Recovered | offered oseltamivir | commercial dairy farm worker | Colorado, USA |  |  | ^12^ |
| unknown | July 2024 | >18 | PCR positive | conjunctivitis and mild influenza-like illness symptoms | Recovered | offered oseltamivir | performed disposal and culling at a poultry farm where HPAI H5N1 had been detected | Colorado, USA | genotype B3.13 |  | ^12^ |
| unknown | July 2024 | >18 | PCR positive | conjunctivitis and mild influenza-like illness symptoms | Recovered | offered oseltamivir | performed disposal and culling at a poultry farm where HPAI H5N1 had been detected | Colorado, USA | genotype B3.13 |  | ^12^ |
| unknown | July 2024 | >18 | PCR positive | conjunctivitis and mild influenza-like illness symptoms | Recovered | offered oseltamivir | performed disposal and culling at a poultry farm where HPAI H5N1 had been detected | Colorado, USA | genotype B3.13 |  | ^12^ |
| unknown | July 2024 | >18 | PCR positive | conjunctivitis and mild influenza-like illness symptoms | Recovered | offered oseltamivir | performed disposal and culling at a poultry farm where HPAI H5N1 had been detected | Colorado, USA | genotype B3.13 |  | ^12^ |
| unknown | July 2024 | >18 | PCR positive | symptoms present but not described; mild illness | Recovered | offered oseltamivir | performed disposal and culling at a poultry farm where HPAI H5N1 had been detected | Colorado, USA | genotype B3.13 |  | ^13^ |
| unknown | July 2024 | >18 | PCR positive | symptoms present but not described; mild illness | Recovered | offered oseltamivir | performed disposal and culling at a poultry farm where HPAI H5N1 had been detected | Colorado, USA | genotype B3.13 |  | ^13^ |
| unknown | July 2024 | >18 | PCR positive | symptoms present but not described; mild illness | Recovered | offered oseltamivir | performed disposal and culling at a poultry farm where HPAI H5N1 had been detected | Colorado, USA |  |  | ^13^ |
| unknown | July 2024 | >18 | PCR positive | symptoms present but not described; mild illness | Recovered | offered oseltamivir | performed disposal and culling at a poultry farm where HPAI H5N1 had been detected | Colorado, USA |  |  | ^13^ |
| unknown | July 2024 | >18 | PCR positive | symptoms present but not described; mild illness | Recovered | offered oseltamivir | performed disposal and culling at a poultry farm where HPAI H5N1 had been detected | Colorado, USA |  |  | ^13^ |
| unknown | August 2024 | >18 | laboratory confirmed, PCR positive nasophayngeal swab specimen | chest pain, nausea, vomiting, diarrhea, weakness | Recovered | hospitalized and treated with oseltamivir | Unknown. Patient reported no contact with animals within 10 days prior to showing symptoms | Missouri, USA | A/Missouri/121/2024 | had history of "severe underlying disease". One close contact developed cough and tiredness on the same day the patient did, but this individual was not tested, had no contact with animals, and recovered without seeking medical care. Six healthcare workers who came into contact with this patient developed mild respiratory symptoms but did not test positive for H5N1 (all tested negative in PCR and antibody testing) | ^13^ |
| unknown | September to October 2024 | >18 | partial or whole genome sequencing | mild illness including conjunctivitis, possibly fever, cough, shortness of breath, and body aches | Recovered | cases treated according to CDC guidelines | commercial dairy farm worker | California, USA | genotype B3.13 |  | ^14^ |
| unknown | September to October 2024 | >18 | partial or whole genome sequencing | mild illness including conjunctivitis, possibly fever, cough, shortness of breath, and body aches | Recovered | cases treated according to CDC guidelines | commercial dairy farm worker | California, USA | genotype B3.13 |  | ^14^ |
| unknown | September to October 2024 | >18 | partial or whole genome sequencing | mild illness including conjunctivitis, possibly fever, cough, shortness of breath, and body aches | Recovered | cases treated according to CDC guidelines | commercial dairy farm worker | California, USA | genotype B3.13 |  | ^14^ |
| unknown | September to October 2024 | >18 | partial or whole genome sequencing | mild illness including conjunctivitis, possibly fever, cough, shortness of breath, and body aches | Recovered | cases treated according to CDC guidelines | commercial dairy farm worker | California, USA | genotype B3.13 |  | ^14^ |
| unknown | September to October 2024 | >18 | partial or whole genome sequencing | mild illness including conjunctivitis, possibly fever, cough, shortness of breath, and body aches | Recovered | cases treated according to CDC guidelines | commercial dairy farm worker | California, USA | genotype B3.13 |  | ^14^ |
| unknown | September to October 2024 | >18 | partial or whole genome sequencing | mild illness including conjunctivitis, possibly fever, cough, shortness of breath, and body aches | Recovered | cases treated according to CDC guidelines | commercial dairy farm worker | California, USA | genotype B3.13 |  | ^14^ |
| unknown | September to October 2024 | >18 | partial or whole genome sequencing | mild illness including conjunctivitis, possibly fever, cough, shortness of breath, and body aches | Recovered | cases treated according to CDC guidelines | commercial dairy farm worker | California, USA | genotype B3.13 |  | ^14^ |
| unknown | September to October 2024 | >18 | partial or whole genome sequencing | mild illness including conjunctivitis, possibly fever, cough, shortness of breath, and body aches | Recovered | cases treated according to CDC guidelines | commercial dairy farm worker | California, USA | genotype B3.13 |  | ^14^ |
| unknown | September to October 2024 | >18 | partial or whole genome sequencing | mild illness including conjunctivitis, possibly fever, cough, shortness of breath, and body aches | Recovered | cases treated according to CDC guidelines | commercial dairy farm worker | California, USA | genotype B3.13 |  | ^14^ |
| unknown | September to October 2024 | >18 | partial or whole genome sequencing | mild illness including conjunctivitis, possibly fever, cough, shortness of breath, and body aches | Recovered | cases treated according to CDC guidelines | commercial dairy farm worker | California, USA |  |  | ^14^ |
| unknown | September to October 2024 | >18 | partial or whole genome sequencing | mild illness including conjunctivitis, possibly fever, cough, shortness of breath, and body aches | Recovered | cases treated according to CDC guidelines | commercial dairy farm worker | California, USA |  |  | ^14^ |
| unknown | September to October 2024 | >18 | partial or whole genome sequencing | mild illness including conjunctivitis, possibly fever, cough, shortness of breath, and body aches | Recovered | cases treated according to CDC guidelines | commercial dairy farm worker | California, USA |  |  | ^14^ |
| unknown | September to October 2024 | >18 | partial or whole genome sequencing | mild illness including conjunctivitis, possibly fever, cough, shortness of breath, and body aches | Recovered | cases treated according to CDC guidelines | commercial dairy farm worker | California, USA |  |  | ^14^ |
| unknown | September to October 2024 | >18 | partial or whole genome sequencing | mild illness including conjunctivitis, possibly fever, cough, shortness of breath, and body aches | Recovered | cases treated according to CDC guidelines | commercial dairy farm worker | California, USA |  |  | ^14^ |
| unknown | September to October 2024 | >18 | partial or whole genome sequencing | mild illness including conjunctivitis, possibly fever, cough, shortness of breath, and body aches | Recovered | cases treated according to CDC guidelines | commercial dairy farm worker | California, USA |  |  | ^14^ |
| unknown | September to October 2024 | >18 | partial or whole genome sequencing | mild illness including conjunctivitis, possibly fever, cough, shortness of breath, and body aches | Recovered | cases treated according to CDC guidelines | commercial dairy farm worker | California, USA |  |  | ^14^ |
| unknown | September to October 2024 | >18 |  | mild illness including conjunctivitis, possibly fever, cough, shortness of breath, and body aches | Recovered | cases treated according to CDC guidelines | commercial dairy farm worker | California, USA |  |  | ^14^ |
| unknown | September to October 2024 | >18 |  | mild illness including conjunctivitis, possibly fever, cough, shortness of breath, and body aches | Recovered | cases treated according to CDC guidelines | commercial dairy farm worker | California, USA |  |  | ^14^ |
| unknown | September to October 2024 | >18 |  | mild illness including conjunctivitis, possibly fever, cough, shortness of breath, and body aches | Recovered | cases treated according to CDC guidelines | commercial dairy farm worker | California, USA |  |  | ^14^ |
| unknown | September to October 2024 | >18 |  | mild illness including conjunctivitis, possibly fever, cough, shortness of breath, and body aches | Recovered | cases treated according to CDC guidelines | commercial dairy farm worker | California, USA |  |  | ^14^ |
| unknown | September to October 2024 | >18 | partial genome sequencing | mild illness; conjunctivitis, cough, muscle aches | Recovered |  | exposure to poultry infected with AIV | Washington, USA | D1.1 genotype |  | ^14^ |
| unknown | September to October 2024 | >18 | partial genome sequencing | mild illness; conjunctivitis | Recovered |  | exposure to poultry infected with AIV | Washington, USA | D1.1 genotype |  | ^14^ |
| unknown | September to October 2024 | >18 | partial genome sequencing | mild illness | Recovered |  | exposure to poultry infected with AIV | Washington, USA | D1.1 genotype |  | ^14^ |
| unknown | September to October 2024 | >18 |  | mild illness | Recovered |  | exposure to poultry infected with AIV | Washington, USA | D1.1 genotype |  | ^14^ |
| unknown | September to October 2024 | >18 |  | mild illness | Recovered |  | exposure to poultry infected with AIV | Washington, USA | D1.1 genotype |  | ^14^ |
| unknown | September to October 2024 | >18 |  | mild illness | Recovered |  | exposure to poultry infected with AIV | Washington, USA | D1.1 genotype |  | ^14^ |
| unknown | September to October 2024 | >18 |  | mild illness | Recovered |  | exposure to poultry infected with AIV | Washington, USA | D1.1 genotype |  | ^14^ |
| unknown | September to October 2024 | >18 |  | mild illness | Recovered |  | exposure to poultry infected with AIV | Washington, USA | D1.1 genotype |  | ^14^ |
| unknown | September to October 2024 | >18 |  | mild illness | Recovered |  | exposure to poultry infected with AIV | Washington, USA | D1.1 genotype |  | ^14^ |
| unknown | September to October 2024 | >18 |  | mild illness | Recovered |  | exposure to poultry infected with AIV | Washington, USA | D1.1 genotype |  | ^14^ |
| unknown | September to October 2024 | >18 |  | mild illness | Recovered |  | exposure to poultry infected with AIV | Washington, USA | D1.1 genotype |  | ^14^ |
| unknown | November to December 2024 | >18 |  | mild illness | Recovered |  | worker at commercial dairy farm with infected cows | California, USA |  |  | ^15^ |
| unknown | November to December 2024 | >18 |  | mild illness | Recovered |  | worker at commercial dairy farm with infected cows | California, USA |  |  | ^15^ |
| unknown | November to December 2024 | >18 |  | mild illness | Recovered |  | worker at commercial dairy farm with infected cows | California, USA |  |  | ^15^ |
| unknown | November to December 2024 | >18 |  | mild illness | Recovered |  | worker at commercial dairy farm with infected cows | California, USA |  |  | ^15^ |
| unknown | November to December 2024 | >18 |  | mild illness | Recovered |  | worker at commercial dairy farm with infected cows | California, USA |  |  | ^15^ |
| unknown | November to December 2024 | >18 |  | mild illness | Recovered |  | worker at commercial dairy farm with infected cows | California, USA |  |  | ^15^ |
| unknown | November to December 2024 | >18 |  | mild illness | Recovered |  | worker at commercial dairy farm with infected cows | California, USA |  |  | ^15^ |
| unknown | November to December 2024 | >18 |  | mild illness | Recovered |  | worker at commercial dairy farm with infected cows | California, USA |  |  | ^15^ |
| unknown | November to December 2024 | >18 |  | mild illness | Recovered |  | worker at commercial dairy farm with infected cows | California, USA |  |  | ^15^ |
| unknown | November to December 2024 | >18 |  | mild illness | Recovered |  | worker at commercial dairy farm with infected cows | California, USA |  |  | ^15^ |
| unknown | November to December 2024 | >18 |  | mild illness | Recovered |  | worker at commercial dairy farm with infected cows | California, USA |  |  | ^15^ |
| unknown | November to December 2024 | <18 | sequencing | mild illness | Recovered |  | No clear source of direct or indirect exposure | California, USA |  | no household contacts tested positive for IAV | ^15^ |
| unknown | November to December 2024 | >18 |  | mild illness | Recovered |  | worker at facilities where H5N1 was detected in poultry; involved with poultry culling and decontamination | Oregon, USA |  |  | ^15^ |
| unknown | November to December 2024 | >18 |  | mild illness | Recovered |  | worker at facilities where H5N1 was detected in poultry; involved with poultry culling and decontamination | Washington, USA | D1.1 genotype |  | ^15^ |
| female | November 2024 | <18 | nasopharyngeal swab tested positive for H5N1 | Conjuctivitis, respriatory symptoms, to critical condition with Acute Respiratory Distress Syndrome | Recovered | antivirals, hospitalized | Unknown with no underlying conditions or travel history; source of infection has not been identified | BC, Canada | D1.1 genotype; had PB2 E627K, HA E186D, HA Q222H mutations | no additional cases, all test from humans and animals and environmental samples were negative | ^15,16^ |
| male | December 2024 | >65 | two positive clinical samples | critical condition with pneumonia | Died | antiviral treatment, hospitalized | owned backyard poultry, had noted deaths in domestic and wild birds prior to symptoms | Louisiana, USA | D1.1 genotype | patient had underlying conditions | ^17^ |
| unknown | December 2024 | >18 | laboratory confirmed | symptoms present but not described; not hospitalized | Recovered |  | worked at poultry facilities | Iowa, USA |  |  | ^17^ |
| unknown | December 2024 | >18 | laboratory confirmed | symptoms present but not described; not hospitalized | Recovered |  | worked at poultry facilities | Wisconsin, USA |  |  | ^17^ |
| unknown | January 2025 | <18 | laboratory confirmed | symptoms present but not described except as mild illness; not hospitalized | Recovered |  | no known contact with infected animals or humans | California, USA |  |  | ^17^ |
| unknown | December 2024 | >18 |  | mild symptoms | Recovered |  | worked at commercial dairy farms where virus was detected | California, USA |  |  | ^17^ |
| unknown | December 2024 | >18 |  | mild symptoms | Recovered |  | worked at commercial dairy farms where virus was detected | California, USA |  |  | ^17^ |
| unknown | December 2024 | >18 |  | mild symptoms | Recovered |  | worked at commercial dairy farms where virus was detected | California, USA |  |  | ^17^ |
| unknown | December 2024 | >18 |  | mild symptoms | Recovered |  | worked at commercial dairy farms where virus was detected | California, USA |  |  | ^17^ |
| unknown | December 2024 | >18 |  | mild symptoms | Recovered |  | worked at commercial dairy farms where virus was detected | California, USA |  |  | ^17^ |
| unknown | January 2025 | unknown | H5 positive | symptomatic but symptoms were not disclosed | Recovered |  | working at a farm where birds were infected with H5N1 | UK | D1.2 genotype | one symptomatic household contact tested negative | ^18^ |
| male | January 2025 | >18 | laboratory confirmed | respiratory and non-respiratory symptoms | Recovered | hospitalized | worked at a commercial poultry facility where H5N1 had been detected, involved with depopulation | Ohio, USA | D1.3 genotype |  | ^18^ |
| female | January to February 2025 | >18 | laboratory confirmed; URT specimens were negative but LRT were positive | altered mental status, weakness, dehydration, fever, chills, cough, pneumonia | Recovered | hospitalized | direct contact with infected poultry that had died on their property | Wyoming, USA | D1.1 genotype; contained PB2 E627K mutation | had underlying health conditions | ^18^ |
| unknown | January 2025 | >18 | laboratory confirmed | conjunctivitis | Recovered | not provided with oseltamivir | worked at commercial dairy farm where virus was detected | Nevada, USA | D1.1 genotype; contained PB2 D701N mutation |  | ^18^ |
| female | April 2025 | 3 years old | Nasopharyngeal swab PCR positive for IAV, H5N1 | respiratory failure | Died | hospitalized | Source of infection unknown | Durango, Mexico | D1.1 genotype | No underlying medical conditions and no history of travel. URT samples from the patient's contacts were negative for H5N1 | ^19^ |

e-Table 1 - Reported human cases of clade 2.3.4.4b H5N1 avian influenza. Exact age and gender were not reported publicly for the majority of cases. Of cases with known patient age ranges, only 3.6% were under 18. This is substantially lower than the historical percentage of cases (55% between the ages of 0 and 19), and is likely due to workplace exposure from poultry and dairy farms being a primary route of transmission.^20^ Three reported cases (3.4%) were fatal, a substantially lower fatality rate than historic H5N1 outbreaks. Of these cases 32 (36.8%) presented with conjunctivitis, 37 (42.5%) had respiratory symptoms, 22 (25.3%) were febrile, 2 (2.3%) had gastrointestinal symptoms, and 6 (6.9%) were asymptomatic. From the available descriptions, it appears respiratory disease has been less frequent in cases associated with the current outbreak compared to historical case series, however, this is difficult to confirm without more detailed descriptions of symptoms for all cases.^21^ For a visualized version of the geographical data within the US, readers can reference the US CDC’s H5 Bird Flu: Current Situation page.^22^

1. Oliver I, Roberts J, Brown CS, et al. A case of avian influenza A(H5N1) in England, January 2022. *Eurosurveillance* 2022;27(5):2200061.

2. State health officials investigate a detection of H5 influenza virus in a human in Colorado | Colorado Department of Public Health and Environment [Internet]. [cited 2025 Aug 13];Available from: https://cdphe.colorado.gov/press-release/state-health-officials-investigate-a-detection-of-h5-influenza-virus-in-a-human

3. Avian Influenza A (H5N1) – Spain [Internet]. [cited 2024 Jan 17];Available from: https://www.who.int/emergencies/disease-outbreak-news/item/2022-DON420

4. Assessment of risk associated with recent influenza A(H5N1) clade 2.3.4.4b viruses [Internet]. [cited 2025 Aug 15];Available from: https://www.who.int/publications/m/item/assessment-of-risk-associated-with-recent-influenza-a(h5n1)-clade-2.3.4.4b-viruses

5. Human infection caused by avian influenza A(H5) - Ecuador [Internet]. [cited 2025 Aug 15];Available from: https://www.who.int/emergencies/disease-outbreak-news/item/2023-DON434

6. WHO. Influenza at the human-animal interface - Summary and risk assessment, from 27 January to 3 March 2023 [Internet]. 2023;Available from: https://cdn.who.int/media/docs/default-source/global-influenza-programme/influenza-at-the-human-animal-interface-summary-and-assessment--from-27-january-to-3-march-2023.pdf?sfvrsn=6065458a_1&download=true

7. WHO. Influenza at the human-animal interface - Summary and risk assessment, from 4 March to 24 April 2023 [Internet]. 2023;Available from: https://cdn.who.int/media/docs/default-source/influenza/human-animal-interface-risk-assessments/influenza-at-the-human-animal-interface-summary-and-assessment--from-4-march-to-24-april-2023.pdf?sfvrsn=e667a5dc_1&download=true

8. Avian Influenza A H5N1 - United Kingdom of Great Britain and Northern Ireland [Internet]. [cited 2025 Aug 15];Available from: https://www.who.int/emergencies/disease-outbreak-news/item/2023-DON468

9. WHO. Influenza at the human-animal interface - Summary and risk assessment, from 1 June to 14 July 2023 [Internet]. 2023;Available from: https://cdn.who.int/media/docs/default-source/influenza/human-animal-interface-risk-assessments/influenza-at-the-human-animal-interface-summary-and-assessment--from-1-june-to-14-july-2023.pdf?sfvrsn=42692eec_1&download=true

10. WHO. Influenza at the human-animal interface - Summary and risk assessment, from 29 March to 3 May 2024 [Internet]. 2024;Available from: https://cdn.who.int/media/docs/default-source/influenza/human-animal-interface-risk-assessments/influenza-at-the-human-animal-interface-summary-and-assessment--from-29-march-to-3-may-2024.pdf?sfvrsn=e9ab83e2_3&download=true

11. WHO. Influenza at the human-animal interface - Summary and risk assessment, from 4 May to 7 June 2024 [Internet]. 2024;Available from: https://cdn.who.int/media/docs/default-source/2021-dha-docs/influenza-at-the-human-animal-interface-summary-and-assessment--from-4-may-to-7-june-2024.pdf?sfvrsn=48c2de05_3&download=true

12. WHO. Influenza at the human-animal interface - Summary and risk assessment, from 8 June to 19 July 2024 [Internet]. 2024;Available from: https://cdn.who.int/media/docs/default-source/influenza/human-animal-interface-risk-assessments/influenza-at-the-human-animal-interface-summary-and-assessment--from-8-june-to-19-july-2024.pdf?sfvrsn=3ebd42dc_3&download=true

13. WHO. Influenza at the human-animal interface - Summary and risk assessment, from 20 July to 27 September 2024 [Internet]. 2024;Available from: https://cdn.who.int/media/docs/default-source/influenza/human-animal-interface-risk-assessments/influenza-at-the-human-animal-interface-summary-and-assessment--from-20-july-to-27-september-2024.pdf?sfvrsn=355e503c_1&download=true

14. WHO. Influenza at the human-animal interface - Summary and risk assessment, from 28 September to 1 November 2024 [Internet]. 2024;Available from: https://cdn.who.int/media/docs/default-source/influenza/human-animal-interface-risk-assessments/influenza-at-the-human-animal-interface-summary-and-assessment--from-28-september-to-1-november-2024.pdf?sfvrsn=1cc12c69_7&download=true

15. WHO. Influenza at the human-animal interface - Summary and risk assessment, from 2 November to 12 December 2024 [Internet]. 2024;Available from: https://cdn.who.int/media/docs/default-source/influenza/human-animal-interface-risk-assessments/influenza_summary_ira_ha_interface_dec.pdf?sfvrsn=b20df48c_1&download=true

16. Jassem AN, Roberts A, Tyson J, et al. Critical Illness in an Adolescent with Influenza A(H5N1) Virus Infection. *N Engl J Med* [Internet] 2024 [cited 2025 Jan 25];Available from: https://www.nejm.org/doi/full/10.1056/NEJMc2415890

17. WHO. Influenza at the human-animal interface - Summary and risk assessment, from 13 December 2024 to 20 January 2025 [Internet]. 2025;Available from: https://cdn.who.int/media/docs/default-source/influenza/human-animal-interface-risk-assessments/influenza-at-the-human-animal-interface-summary-and-assessment--from-13-december-2024-to-20-january-2025.pdf?sfvrsn=aff4e6b9_3&download=true

18. WHO. Influenza at the human-animal interface - Summary and risk assessment, from 20 January to 19 March 2025 [Internet]. 2025;Available from: https://cdn.who.int/media/docs/default-source/influenza/human-animal-interface-risk-assessments/influenza-at-the-human-animal-interface-summary-and-assessment--from-21-january-to-19-march-2025o-xx.pdf?sfvrsn=dba96c6c_9&download=true

19. WHO. Influenza at the human-animal interface - Summary and risk assessment, from 20 March to 22 April 2025 [Internet]. 2025;Available from: https://cdn.who.int/media/docs/default-source/influenza/human-animal-interface-risk-assessments/influenza-at-the-human-animal-interface-summary-and-assessment--from-20-march-to-22-april-2025.pdf?sfvrsn=94dbbd93_3&download=true

20. Smallman-Raynor M, Cliff AD. Avian Influenza A (H5N1) Age Distribution in Humans. *Emerg Infect Dis* 2007;13(3):510–512.

21. Korteweg C, Gu J. Pathology, Molecular Biology, and Pathogenesis of Avian Influenza A (H5N1) Infection in Humans. *Am J Pathol* 2008;172(5):1155–1170.

22. CDC. H5 Bird Flu: Current Situation [Internet]. Avian Influenza Bird Flu. 2025 [cited 2025 Aug 13];Available from: https://www.cdc.gov/bird-flu/situation-summary/index.html
