# Supplementary material for: Examining the Threat of H5N1 Highly Pathogenic Avian Influenza to Human Health
Source: Chest. 2025 Nov 4;169(4):947–57. doi: 10.1016/j.chest.2025.10.030 (PMC13084735; doi:10.1016/j.chest.2025.10.030)
Supplement: e-Online Data [file mmc2.docx]

**Examining the threat of H5N1 highly pathogenic avian influenza to human health**

Authors: Juliette Blais-Savoie, BSc^1,2^, Emily Halajian, MSc^1,2^, Kuganya Nirmalarajah, BHSc^1,2^, Andra Banete, PhD^1^, Juan C. Corredor, PhD^1^, Jonathon D. Kotwa, PhD^1^, Yaejin Lee, BSc^1,2^, Sugandha Raj, PhD^3^, Shayan Sharif, PhD DVM^3^, Nicole Mideo PhD^4^, Samira Mubareka, MD^1,2^

**Supplemental Material (e-Table 2)**

| **Common name** | **Scientific name** | **Captive/Wild** | **Locations** | **Mammalian adaptations (PB2)** |
| --- | --- | --- | --- | --- |
| Arctic fox | *Vulpes lagopus* | Captive | Finland[^1^](https://www.zotero.org/google-docs/?pRRJnI) | ·· |
| Grey fox | *Urocyon cinereoargenteus* | Wild | USA[^2^](https://www.zotero.org/google-docs/?CYJxE6) | ·· |
| Red fox | *Vulpes vulpes* | Captive, wild | Belgium,[^3^](https://www.zotero.org/google-docs/?ulA3Ho) Canada,[^4^](https://www.zotero.org/google-docs/?vObWgL)  England,[^5^](https://www.zotero.org/google-docs/?WkaBFk) Estonia,[^5^](https://www.zotero.org/google-docs/?vmgGUY)  Finland,[^5^](https://www.zotero.org/google-docs/?ugJEzy) France,[^6^](https://www.zotero.org/google-docs/?cJ1d2Q) Germany,[^7^](https://www.zotero.org/google-docs/?3wvDhe) Japan,[^8^](https://www.zotero.org/google-docs/?KshUy5) Latvia,[^9^](https://www.zotero.org/google-docs/?U0JnXN) Netherlands,[^10^](https://www.zotero.org/google-docs/?BYtpn6) Northern Ireland,[^11^](https://www.zotero.org/google-docs/?eG4gjC) Norway,[^12^](https://www.zotero.org/google-docs/?YH3kgJ) Scotland,[^13^](https://www.zotero.org/google-docs/?apKrRP) Slovenia,[^14^](https://www.zotero.org/google-docs/?HwpI4p) Sweden,[^5^](https://www.zotero.org/google-docs/?qoCDqK) USA,[^15^](https://www.zotero.org/google-docs/?iLcOUK) Wales[^13^](https://www.zotero.org/google-docs/?cF4SHD) | E627K, E627V, D701N, T271A |
| Coyote | *Canis latrans* | Wild | USA[^15^](https://www.zotero.org/google-docs/?zG7XQ3) | D701N |
| Domestic dog | *Canis familiaris* | Captive | Canada,[^16^](https://www.zotero.org/google-docs/?I9lvZU) Italy,[^17^](https://www.zotero.org/google-docs/?yvug1F) Poland[^18^](https://www.zotero.org/google-docs/?JRfJ75) | ·· |
| Raccoon dog, tanuki | *Nyctereutes procyonoides* | Captive, wild | Finland,[^1^](https://www.zotero.org/google-docs/?iBUk6q) Japan[^8^](https://www.zotero.org/google-docs/?JAk40E) | ·· |
| South American bush dog | *Speothos venaticus* | Captive | England[^13^](https://www.zotero.org/google-docs/?ildWJ6) | ·· |
| Abert's squirrel | *Sciurus aberti* | Wild | USA[^15^](https://www.zotero.org/google-docs/?CJpo1a) | ·· |
| American black bear | *Ursus americanus* | Wild | Canada,[^19^](https://www.zotero.org/google-docs/?KNK4Um) USA[^15^](https://www.zotero.org/google-docs/?U8R6QH) | D701N |
| Asiatic black bear | *Ursus thibetanus* | Captive | France[^20^](https://www.zotero.org/google-docs/?tWQucK) | E627K |
| Brown/Grizzly bear | *Ursus arctos* | Captive, wild | USA[^15^](https://www.zotero.org/google-docs/?hri9Io) | ·· |
| Polar bear | *Ursus maritimus* | Wild | USA[^15^](https://www.zotero.org/google-docs/?pkLplF) | ·· |
| Lion | *Panthera leo* | Captive | Peru[^21^](https://www.zotero.org/google-docs/?CipYcL), USA[^15^](https://www.zotero.org/google-docs/?NVplEp) | ·· |
| Tiger | *Panthera tigris* | Captive | USA[^15^](https://www.zotero.org/google-docs/?BSJJk9) | ·· |
| Leopard | *Panthera pardus* | Captive | USA[^15^](https://www.zotero.org/google-docs/?dsp7nI) | ·· |
| Bobcat | *Lynx rufus* | Wild | USA[^15^](https://www.zotero.org/google-docs/?bJZ71P) | ·· |
| Serval | *Leptailurus serval* | Captive | USA[^22^](https://www.zotero.org/google-docs/?9hbSst) | ·· |
| Geoffroy’s cat | *Leopardus geoffroyi* | Captive | USA[^22^](https://www.zotero.org/google-docs/?r6lqD3) | ·· |
| Domestic cat | *Felis catus* | Captive | Canada[^23(p)^](https://www.zotero.org/google-docs/?wbAKUj), France,[^24^](https://www.zotero.org/google-docs/?sNIn9O) Hungary[^25^](https://www.zotero.org/google-docs/?IFDPC3), Italy,[^17^](https://www.zotero.org/google-docs/?VFBhiW) Poland,[^26^](https://www.zotero.org/google-docs/?MqPo8x) South Korea,[^27^](https://www.zotero.org/google-docs/?3mCVAq) USA[^15^](https://www.zotero.org/google-docs/?A5K9rX) | E627K |
| European polecat | *Mustela putorius* | Wild | Belgium,[^3^](https://www.zotero.org/google-docs/?QGy8Zh) Netherlands[^28^](https://www.zotero.org/google-docs/?VPGAg7) | E627K, T271A |
| Puma | *Puma concolor* | Captive, wild | USA[^15^](https://www.zotero.org/google-docs/?B4b4a2) | ·· |
| Canada Lynx | *Lynx canadensis* | Captive | USA[^15^](https://www.zotero.org/google-docs/?aQKMfH) | ·· |
| Eurasian Lynx | *Lynx lynx* | Captive, wild | Finland[^1^](https://www.zotero.org/google-docs/?EWfd2V), USA[^15^](https://www.zotero.org/google-docs/?fwYeQA) | E627K, D701N |
| Tiger | *Panthera tigris* | Captive | USA[^15^](https://www.zotero.org/google-docs/?VfwSVi) | ·· |
| Atlantic white-sided dolphin | *Lagenorhynchus acutus* | Wild | Canada[^29^](https://www.zotero.org/google-docs/?B05EO8) | ·· |
| Bottlenose dolphin | *Tursiops truncatus* | Wild | USA[^15^](https://www.zotero.org/google-docs/?GASNdE) | ·· |
| Chilean dolphin | *Cephalorhynchus eutropia* | Wild | Chile[^30^](https://www.zotero.org/google-docs/?LYquDv) | ·· |
| Common dolphin | *Delphinus delphis* | Wild | England,[^13^](https://www.zotero.org/google-docs/?7JLDyy) Peru,[^31^](https://www.zotero.org/google-docs/?pqtLWH) Wales[^13^](https://www.zotero.org/google-docs/?LZARR3) | ·· |
| Antarctic fur seal | *Arctocephalus gazella* | Wild | South Georgia[^32^](https://www.zotero.org/google-docs/?iHDnBS) | ·· |
| Caspian seal | *Pusa caspica* | Wild | Russia[^33^](https://www.zotero.org/google-docs/?Lexrwh) | ·· |
| Grey seal | *Halichoerus grypus* | Wild | Canada,[^34^](https://www.zotero.org/google-docs/?z7s7gk) England,[^13^](https://www.zotero.org/google-docs/?lne2on) Germany,[^35^](https://www.zotero.org/google-docs/?Q18Cvo) Netherlands,[^35^](https://www.zotero.org/google-docs/?zs18Tt) Scotland,[^13^](https://www.zotero.org/google-docs/?yudLEZ) USA[^15^](https://www.zotero.org/google-docs/?ubWsDI) | E627K |
| Northern fur seal | *Callorhinus ursinus* | Wild | Russia[^36^](https://www.zotero.org/google-docs/?ZcEu31) | ·· |
| Harbor seal | *Phoca vitulina* | Wild | Canada,[^34^](https://www.zotero.org/google-docs/?YjlQ6v) Denmark,[^37^](https://www.zotero.org/google-docs/?uBvi96) Scotland,[^13^](https://www.zotero.org/google-docs/?EMI6CC) USA[^15(p63)^](https://www.zotero.org/google-docs/?ehvkzo) | E627K, D701N |
| Southern elephant seal | *Mirounga leonina* | Wild | Argentina,[^38^](https://www.zotero.org/google-docs/?CNZnEP) South Georgia[^32^](https://www.zotero.org/google-docs/?FJU4ES) | ·· |
| Southern fur seal | *Arctocephalus australis* | Wild | Brazil[^39^](https://www.zotero.org/google-docs/?59sZsa) | ·· |
| South American sea lion | *Otaria flavescens* | Wild | Argentina,[^40^](https://www.zotero.org/google-docs/?7nVznX) Brazil,[^40^](https://www.zotero.org/google-docs/?C2OHwg) Chile,[^41^](https://www.zotero.org/google-docs/?L0RZsR) Peru,[^42^](https://www.zotero.org/google-docs/?BfYhqX) Uruguay[^40^](https://www.zotero.org/google-docs/?G2GZDj) | D701N |
| Burmeister's porpoise | *Phocoena spinipinnis* | Wild | Chile[^41^](https://www.zotero.org/google-docs/?GRiwaZ) | ·· |
| Harbor porpoise | *Phocoena phocoena* | Wild | Sweden,[^43^](https://www.zotero.org/google-docs/?sA0tKb) England[^13^](https://www.zotero.org/google-docs/?jL0M8N) | ·· |
| Eurasian otter | *Lutra lutra* | Wild | England,[^13^](https://www.zotero.org/google-docs/?mD520S)  Estonia,[^5^](https://www.zotero.org/google-docs/?TixAy6) Finland,[^44^](https://www.zotero.org/google-docs/?B9EmmG) Netherlands,[^45^](https://www.zotero.org/google-docs/?nnKadU) Scotland,[^13^](https://www.zotero.org/google-docs/?LVEg3K)  Sweden[^5^](https://www.zotero.org/google-docs/?wBLg45) | E627K |
| North American river otter | *Lontra canadensis* | Wild | USA[^15^](https://www.zotero.org/google-docs/?kQCBck) | ·· |
| Marine otter | *Lontra felina* | Wild | Chile[^41^](https://www.zotero.org/google-docs/?3P8fRE) | ·· |
| South American river otter | *Lontra provocax* | Wild | Chile[^41^](https://www.zotero.org/google-docs/?93HR8b) | ·· |
| European badger | *Meles meles* | Wild | Netherlands[^10^](https://www.zotero.org/google-docs/?VGadhK) | ·· |
| American mink | *Neovison vison* | Captive, wild | Canada,[^4^](https://www.zotero.org/google-docs/?P5qqOf) Finland,[^46^](https://www.zotero.org/google-docs/?r7Njoj) Spain[^47^](https://www.zotero.org/google-docs/?puqJEx) | T271A |
| Ferret | *Mustela furo* | Captive | Belgium,[^3^](https://www.zotero.org/google-docs/?UPT17c) Poland,[^48^](https://www.zotero.org/google-docs/?8UwjyV) Slovenia[^5^](https://www.zotero.org/google-docs/?JjKpBF) | ·· |
| Fisher | *Pekania pennanti* | Wild | USA[^15^](https://www.zotero.org/google-docs/?N5iX1J) | ·· |
| American marten | *Martes americana* | Wild | USA[^15^](https://www.zotero.org/google-docs/?IWlX57) | ·· |
| Stone marten | *Martes foina* | Wild | Netherlands[^10^](https://www.zotero.org/google-docs/?hqQDP9) | ·· |
| European pine marten | *Martes martes* | Wild | Germany[^49^](https://www.zotero.org/google-docs/?LG7MpN) | ·· |
| Sable | *Martes zibellina* | Captive | Finland[^50^](https://www.zotero.org/google-docs/?kPAROE) | ·· |
| Prairie vole | *Microtus ochrogaster* | Wild | USA[^15^](https://www.zotero.org/google-docs/?N0JbyO) | ·· |
| Desert Cottontail | *Sylvilagus audubonii* | Wild | USA[^51^](https://www.zotero.org/google-docs/?d1ym3K) | ·· |
| Deer mouse | *Peromyscus maniculatus* | Wild | USA[^51^](https://www.zotero.org/google-docs/?7oTWCD) | ·· |
| House mouse | *Mus musculus* | Wild | USA[^15^](https://www.zotero.org/google-docs/?8Pp15T) | ·· |
| Norway Rat | *Rattus norvegicus* | Wild | Egypt[^52^](https://www.zotero.org/google-docs/?t9lrg9) | L89V, G309D, T339K |
| Striped skunk | *Mephitis mephitis* | Wild | Canada,[^4^](https://www.zotero.org/google-docs/?klSKqa) USA[^15^](https://www.zotero.org/google-docs/?nzrP62) | ·· |
| Northern Raccoon | *Procyon lotor* | Wild | Canada,[^4^](https://www.zotero.org/google-docs/?YJ9PnU) Germany,[^49^](https://www.zotero.org/google-docs/?XliCOq) USA[^15^](https://www.zotero.org/google-docs/?8TNPr6) | E627K |
| South American Coati | *Nasua nasua* | Captive, wild | Germany,[^49^](https://www.zotero.org/google-docs/?yQsO3y) Uruguay[^53^](https://www.zotero.org/google-docs/?eYHXog) | ·· |
| Cattle | *Bos taurus* | Captive | USA[^15^](https://www.zotero.org/google-docs/?OfmoBw) | ·· |
| Domestic sheep | *Ovis aries* | Captive | England[^54^](https://www.zotero.org/google-docs/?re8TAv) | ·· |
| Goat | *Capra hircus* | Captive | USA[^15^](https://www.zotero.org/google-docs/?A1mFLV) | ·· |
| Alpaca | *Lama pacos* | Captive | USA[^55^](https://www.zotero.org/google-docs/?z2rIsU) | ·· |
| Domestic pig | *Sus domesticus* | Captive | Italy[^56^](https://www.zotero.org/google-docs/?L5LMNP) | ·· |
| Virginia opossum | *Didelphis virginiana* | Wild | USA[^15^](https://www.zotero.org/google-docs/?cglGLf) | ·· |

e-Table 2 - Mammalian infections with clade 2.3.4.4b H5N1 and associated key PB2 mammalian adaptation mutations.

References

[1. WOAH. *Finland - Influenza A Viruses of High Pathogenicity (Inf. with) (Non-Poultry Including Wild Birds) (2017-) - Follow up Report 39*. Accessed April 12, 2024. https://wahis.woah.org/#/in-review/5119?fromPage=event-dashboard-url](https://www.zotero.org/google-docs/?cyHZaf)

[2. Elsmo EJ, Wünschmann A, Beckmen KB, et al. Highly Pathogenic Avian Influenza A(H5N1) Virus Clade 2.3.4.4b Infections in Wild Terrestrial Mammals, United States, 2022. *Emerg Infect Dis*. 2023;29(12). doi:10.3201/eid2912.230464](https://www.zotero.org/google-docs/?cyHZaf)

[3. WOAH. *Belgium - Influenza A Viruses of High Pathogenicity (Inf. with) (Non-Poultry Including Wild Birds) (2017-) - Follow up Report 6*. Accessed April 15, 2024. https://wahis.woah.org/#/in-review/4971](https://www.zotero.org/google-docs/?cyHZaf)

[4. Alkie TN, Cox S, Embury-Hyatt C, et al. Characterization of neurotropic HPAI H5N1 viruses with novel genome constellations and mammalian adaptive mutations in free-living mesocarnivores in Canada. *Emerg Microbes Infect*. 2023;12(1):2186608. doi:10.1080/22221751.2023.2186608](https://www.zotero.org/google-docs/?cyHZaf)

[5. Adlhoch C, Fusaro A, Gonzales JL, et al. Avian influenza overview September – December 2021. *EFSA J*. 2021;19(12):e07108. doi:10.2903/j.efsa.2021.7108](https://www.zotero.org/google-docs/?cyHZaf)

[6. WAHIS. France - Influenza A viruses of high pathogenicity (Inf. with) (non-poultry including wild birds) (2017-) - Follow up report 1 [FINAL]. Accessed August 14, 2024. https://wahis.woah.org/#/in-review/4941](https://www.zotero.org/google-docs/?cyHZaf)

[7. Baechlein C, Kleinschmidt S, Hartmann D, et al. Neurotropic Highly Pathogenic Avian Influenza A(H5N1) Virus in Red Foxes, Northern Germany. *Emerg Infect Dis*. 2023;29(12):2509-2512. doi:10.3201/eid2912.230938](https://www.zotero.org/google-docs/?cyHZaf)

[8. Hiono T, Kobayashi D, Kobayashi A, et al. Virological, pathological, and glycovirological investigations of an Ezo red fox and a tanuki naturally infected with H5N1 high pathogenicity avian influenza viruses in Hokkaido, Japan. *Virology*. 2023;578:35-44. doi:10.1016/j.virol.2022.11.008](https://www.zotero.org/google-docs/?cyHZaf)

[9. WAHIS. Latvia - Influenza A viruses of high pathogenicity (Inf. with) (non-poultry including wild birds) (2017-) - Follow up report 25 [FINAL]. Accessed August 14, 2024. https://wahis.woah.org/#/in-review/5124?fromPage=event-dashboard-url](https://www.zotero.org/google-docs/?cyHZaf)

[10. Bordes L, Vreman S, Heutink R, et al. Highly Pathogenic Avian Influenza H5N1 Virus Infections in Wild Red Foxes (Vulpes vulpes) Show Neurotropism and Adaptive Virus Mutations. Richard M, ed. *Microbiol Spectr*. 2023;11(1):e02867-22. doi:10.1128/spectrum.02867-22](https://www.zotero.org/google-docs/?cyHZaf)

[11. Lagan P, McKenna R, Baleed S, et al. Highly pathogenic avian influenza A(H5N1) virus infection in foxes with PB2-M535I identified as a novel mammalian adaptation, Northern Ireland, July 2023. *Eurosurveillance*. 2023;28(42). doi:10.2807/1560-7917.ES.2023.28.42.2300526](https://www.zotero.org/google-docs/?cyHZaf)

[12. WAHIS. Norway - Influenza A viruses of high pathogenicity (Inf. with) (non-poultry including wild birds) (2017-) - Follow up report 1 [FINAL]. Accessed August 13, 2024. https://wahis.woah.org/#/in-review/4451?reportId=168571&fromPage=event-dashboard-url](https://www.zotero.org/google-docs/?cyHZaf)

[13. Confirmed findings of influenza of avian origin in non-avian wildlife. GOV.UK. Accessed April 12, 2024. https://www.gov.uk/government/publications/bird-flu-avian-influenza-findings-in-non-avian-wildlife/confirmed-findings-of-influenza-of-avian-origin-in-non-avian-wildlife](https://www.zotero.org/google-docs/?cyHZaf)

[14. WAHIS. Slovenia - Influenza A viruses of high pathogenicity (Inf. with) (non-poultry including wild birds) (2017-) - Follow up report 1 [FINAL]. Accessed April 1, 2025. https://wahis.woah.org/#/in-review/6119?fromPage=event-dashboard-url](https://www.zotero.org/google-docs/?cyHZaf)

[15. WAHIS. United States of America - Influenza A viruses of high pathogenicity (Inf. with) (non-poultry including wild birds) (2017-) - Follow up report 96. April 11, 2024. Accessed April 1, 2025. https://wahis.woah.org/#/in-review/4451?fromPage=event-dashboard-url](https://www.zotero.org/google-docs/?cyHZaf)

[16. Agency CFI. Domestic dog tests positive for avian influenza in Canada. April 4, 2023. Accessed January 8, 2024. https://www.canada.ca/en/food-inspection-agency/news/2023/04/domestic-dog-tests-positive-for-avian-influenza-in-canada.html](https://www.zotero.org/google-docs/?cyHZaf)

[17. Moreno A, Bonfante F, Bortolami A, et al. Asymptomatic infection with clade 2.3.4.4b highly pathogenic avian influenza A(H5N1) in carnivore pets, Italy, April 2023. *Eurosurveillance*. 2023;28(35). doi:10.2807/1560-7917.ES.2023.28.35.2300441](https://www.zotero.org/google-docs/?cyHZaf)

[18. Szaluś-Jordanow O, Golke A, Dzieciątkowski T, et al. Upper Respiratory Tract Disease in a Dog Infected by a Highly Pathogenic Avian A/H5N1 Virus. *Microorganisms*. 2024;12(4):689. doi:10.3390/microorganisms12040689](https://www.zotero.org/google-docs/?cyHZaf)

[19. Jakobek BT, Berhane Y, Nadeau MS, et al. Influenza A(H5N1) Virus Infections in 2 Free-Ranging Black Bears ( *Ursus americanus* ), Quebec, Canada. *Emerg Infect Dis*. 2023;29(10). doi:10.3201/eid2910.230548](https://www.zotero.org/google-docs/?cyHZaf)

[20. Bessière P, Gaide N, Croville G, et al. High pathogenicity avian influenza A (H5N1) clade 2.3.4.4b virus infection in a captive Tibetan black bear (Ursus thibetanus): investigations based on paraffin-embedded tissues, France, 2022. *Microbiol Spectr*. 2024;12(3):e03736-23. doi:10.1128/spectrum.03736-23](https://www.zotero.org/google-docs/?cyHZaf)

[21. Mortality in mammals (Peru) (in Spanish). WOAH - World Organisation for Animal Health. Accessed April 2, 2025. https://www.woah.org/en/document/mortality-in-mammals-peru/](https://www.zotero.org/google-docs/?cyHZaf)

[22. H5N1 sickens Iowa poultry worker; virus strikes more cats, wild birds, and poultry | CIDRAP. December 23, 2024. Accessed April 2, 2025. https://www.cidrap.umn.edu/avian-influenza-bird-flu/h5n1-sickens-iowa-poultry-worker-virus-strikes-more-cats-wild-birds-and](https://www.zotero.org/google-docs/?cyHZaf)

[23. WAHIS. Canada - Influenza A viruses of high pathogenicity (Inf. with) (non-poultry including wild birds) (2017-) - Follow up report 22. Accessed April 2, 2025. https://wahis.woah.org/#/in-review/4438?fromPage=event-dashboard-url](https://www.zotero.org/google-docs/?cyHZaf)

[24. Briand FX, Souchaud F, Pierre I, et al. Highly Pathogenic Avian Influenza A(H5N1) Clade 2.3.4.4b Virus in Domestic Cat, France, 2022. *Emerg Infect Dis*. 2023;29(8). doi:10.3201/eid2908.230188](https://www.zotero.org/google-docs/?cyHZaf)

[25. HPAI in a cat in Hungary. WOAH - World Organisation for Animal Health. Accessed April 2, 2025. https://www.woah.org/en/document/hpai-in-a-cat-in-hungary/](https://www.zotero.org/google-docs/?cyHZaf)

[26. Domańska-Blicharz K, Świętoń E, Świątalska A, et al. Outbreak of highly pathogenic avian influenza A(H5N1) clade 2.3.4.4b virus in cats, Poland, June to July 2023. *Eurosurveillance*. 2023;28(31). doi:10.2807/1560-7917.ES.2023.28.31.2300366](https://www.zotero.org/google-docs/?cyHZaf)

[27. WAHIS. Korea (Rep. of) - Influenza A viruses of high pathogenicity (Inf. with) (non-poultry including wild birds) (2017-) - Follow up report 3 [FINAL]. Accessed August 14, 2024. https://wahis.woah.org/#/in-review/5144?fromPage=event-dashboard-url](https://www.zotero.org/google-docs/?cyHZaf)

[28. Chestakova IV, Van Der Linden A, Bellido Martin B, et al. High number of HPAI H5 virus infections and antibodies in wild carnivores in the Netherlands, 2020–2022. *Emerg Microbes Infect*. 2023;12(2):2270068. doi:10.1080/22221751.2023.2270068](https://www.zotero.org/google-docs/?cyHZaf)

[29. National Avian Influenza - Wild Positives. Accessed August 14, 2024. https://cfia-ncr.maps.arcgis.com/apps/dashboards/89c779e98cdf492c899df23e1c38fdbc](https://www.zotero.org/google-docs/?cyHZaf)

[30. Godoy M, de Oca MM, Caro D, Pontigo JP, Kibenge M, Kibenge F. Evolution and Current Status of Influenza A Virus in Chile: A Review. *Pathogens*. 2023;12(10):1252. doi:10.3390/pathogens12101252](https://www.zotero.org/google-docs/?cyHZaf)

[31. Leguia M, Garcia-Glaessner A, Muñoz-Saavedra B, et al. Highly pathogenic avian influenza A (H5N1) in marine mammals and seabirds in Peru. *Nat Commun*. 2023;14(1):5489. doi:10.1038/s41467-023-41182-0](https://www.zotero.org/google-docs/?cyHZaf)

[32. Bennison A, Byrne AMP, Reid SM, et al. Detection and spread of high pathogenicity avian influenza virus H5N1 in the Antarctic Region. Published online November 24, 2023:2023.11.23.568045. doi:10.1101/2023.11.23.568045](https://www.zotero.org/google-docs/?cyHZaf)

[33. Avian flu strikes more poultry in 6 states as virus found in dead seals in Russia | CIDRAP. January 24, 2023. Accessed April 12, 2024. https://www.cidrap.umn.edu/avian-influenza-bird-flu/avian-flu-strikes-more-poultry-6-states-virus-found-dead-seals-russia](https://www.zotero.org/google-docs/?cyHZaf)

[34. Lair S, Quesnel L, Berhane Y, et al. *Outbreak of Highly Pathogenic Avian Influenza Virus H5N1 in Seals in the St. Lawrence Estuary, Quebec, Canada*. Pathology; 2023. doi:10.1101/2023.11.16.567398](https://www.zotero.org/google-docs/?cyHZaf)

[35. Mirolo M, Pohlmann A, Ahrens AK, et al. Highly pathogenic avian influenza A virus (HPAIV) H5N1 infection in two European grey seals (Halichoerus grypus) with encephalitis. *Emerg Microbes Infect*. 12(2):e2257810. doi:10.1080/22221751.2023.2257810](https://www.zotero.org/google-docs/?cyHZaf)

[36. WAHIS. Russia - Influenza A viruses of high pathogenicity (Inf. with) (non-poultry including wild birds) (2017-) - Follow up report 2 [FINAL]. Accessed August 14, 2024. https://wahis.woah.org/#/in-review/5191?fromPage=event-dashboard-url](https://www.zotero.org/google-docs/?cyHZaf)

[37. Report of HPAI H5N1 in harbor seals in Denmark. WOAH - World Organisation for Animal Health. Accessed August 14, 2024. https://www.woah.org/en/document/report-of-hpai-h5n1-in-harbor-seals-in-denmark/](https://www.zotero.org/google-docs/?cyHZaf)

[38. Campagna C, Uhart M, Falabella V, et al. Catastrophic mortality of southern elephant seals caused by H5N1 avian influenza. *Mar Mammal Sci*. 2024;40(1):322-325. doi:10.1111/mms.13101](https://www.zotero.org/google-docs/?cyHZaf)

[39. WAHIS. Brazil - Influenza A viruses of high pathogenicity (Inf. with) (non-poultry including wild birds) (2017-) - Follow up report 13 [FINAL]. Accessed August 14, 2024. https://wahis.woah.org/#/in-review/5259?fromPage=event-dashboard-url](https://www.zotero.org/google-docs/?cyHZaf)

[40. Plaza PI, Gamarra-Toledo V, Rodríguez Euguí J, Rosciano N, Lambertucci SA. Pacific and Atlantic sea lion mortality caused by highly pathogenic Avian Influenza A(H5N1) in South America. *Travel Med Infect Dis*. 2024;59:102712. doi:10.1016/j.tmaid.2024.102712](https://www.zotero.org/google-docs/?cyHZaf)

[41. García-Cegarra AM, Hall A, Martínez-López E. Bycatch and pollution are the main threats for Burmeister’s porpoises inhabiting a high-industrialized bay in the Humboldt Current System. *Environ Res*. 2024;251:118621. doi:10.1016/j.envres.2024.118621](https://www.zotero.org/google-docs/?cyHZaf)

[42. Gamarra-Toledo V, Plaza PI, Gutiérrez R, et al. Mass Mortality of Sea Lions Caused by Highly Pathogenic Avian Influenza A(H5N1) Virus. *Emerg Infect Dis*. 2023;29(12):2553-2556. doi:10.3201/eid2912.230192](https://www.zotero.org/google-docs/?cyHZaf)

[43. Thorsson E, Zohari S, Roos A, Banihashem F, Bröjer C, Neimanis A. Highly Pathogenic Avian Influenza A(H5N1) Virus in a Harbor Porpoise, Sweden. *Emerg Infect Dis*. 2023;29(4):852-855. doi:10.3201/eid2904.221426](https://www.zotero.org/google-docs/?cyHZaf)

[44. Tammiranta N, Isomursu M, Fusaro A, et al. Highly pathogenic avian influenza A (H5N1) virus infections in wild carnivores connected to mass mortalities of pheasants in Finland. *Infect Genet Evol*. 2023;111:105423. doi:10.1016/j.meegid.2023.105423](https://www.zotero.org/google-docs/?cyHZaf)

[45. Vreman S, Kik M, Germeraad E, et al. Zoonotic Mutation of Highly Pathogenic Avian Influenza H5N1 Virus Identified in the Brain of Multiple Wild Carnivore Species. *Pathogens*. 2023;12(2):168. doi:10.3390/pathogens12020168](https://www.zotero.org/google-docs/?cyHZaf)

[46. Lindh E, Lounela H, Ikonen N, et al. Highly pathogenic avian influenza A(H5N1) virus infection on multiple fur farms in the South and Central Ostrobothnia regions of Finland, July 2023. *Eurosurveillance*. 2023;28(31). doi:10.2807/1560-7917.ES.2023.28.31.2300400](https://www.zotero.org/google-docs/?cyHZaf)

[47. Agüero M, Monne I, Sánchez A, et al. Highly pathogenic avian influenza A(H5N1) virus infection in farmed minks, Spain, October 2022. *Euro Surveill Bull Eur Sur Mal Transm Eur Commun Dis Bull*. 2023;28(3):2300001. doi:10.2807/1560-7917.ES.2023.28.3.2300001](https://www.zotero.org/google-docs/?cyHZaf)

[48. Golke A, Jańczak D, Szaluś-Jordanow O, et al. Natural Infection with Highly Pathogenic Avian Influenza A/H5N1 Virus in Pet Ferrets. *Viruses*. 2024;16(6):931. doi:10.3390/v16060931](https://www.zotero.org/google-docs/?cyHZaf)

[49. WAHIS. Germany - Influenza A viruses of high pathogenicity (Inf. with) (non-poultry including wild birds) (2017-) - Follow up report 5 [Final]. Accessed April 2, 2025. https://wahis.woah.org/#/in-review/5031?reportId=160702&fromPage=event-dashboard-url](https://www.zotero.org/google-docs/?cyHZaf)

[50. WAHIS. Finland - Influenza A viruses of high pathogenicity (Inf. with) (non-poultry including wild birds) (2017-) - Follow up report 41 [FINAL]. Accessed August 14, 2024. https://wahis.woah.org/#/in-review/5119?fromPage=event-dashboard-url](https://www.zotero.org/google-docs/?cyHZaf)

[51. HPAI Detections in Mammals. Accessed August 14, 2024. https://www.aphis.usda.gov/livestock-poultry-disease/avian/avian-influenza/hpai-detections/mammals](https://www.zotero.org/google-docs/?cyHZaf)

[52. Kutkat O, Gomaa ,Mokhtar, Moatasim ,Yassmin, et al. Highly pathogenic avian influenza virus H5N1 clade 2.3.4.4b in wild rats in Egypt during 2023. *Emerg Microbes Infect*. 2024;13(1):2396874. doi:10.1080/22221751.2024.2396874](https://www.zotero.org/google-docs/?cyHZaf)

[53. WAHIS. Uruguay - Influenza A viruses of high pathogenicity (Inf. with) (non-poultry including wild birds) (2017-) - Follow up report 1 [FINAL]. Accessed August 14, 2024. https://wahis.woah.org/#/in-review/5046?fromPage=event-dashboard-url](https://www.zotero.org/google-docs/?cyHZaf)

[54. Influenza of avian origin confirmed in a sheep in Yorkshire. GOV.UK. Accessed April 2, 2025. https://www.gov.uk/government/news/influenza-of-avian-origin-confirmed-in-a-sheep-in-yorkshire](https://www.zotero.org/google-docs/?cyHZaf)

[55. Highly Pathogenic Avian Influenza (HPAI) H5N1 Detections in Alpacas | Animal and Plant Health Inspection Service. Accessed August 2, 2024. https://www.aphis.usda.gov/livestock-poultry-disease/avian/avian-influenza/hpai-detections/mammals/highly-pathogenic-avian](https://www.zotero.org/google-docs/?cyHZaf)

[56. Rosone F, Bonfante F, Sala MG, et al. Seroconversion of a Swine Herd in a Free-Range Rural Multi-Species Farm against HPAI H5N1 2.3.4.4b Clade Virus. *Microorganisms*. 2023;11(5):1162. doi:10.3390/microorganisms11051162](https://www.zotero.org/google-docs/?cyHZaf)
