# Supplementary material for: Examining the Threat of H5N1 Highly Pathogenic Avian Influenza to Human Health
Source: Chest. 2025 Nov 4;169(4):947–57. doi: 10.1016/j.chest.2025.10.030 (PMC13084735; doi:10.1016/j.chest.2025.10.030)
Supplement: e-Online Data [file mmc3.docx]

**Examining the threat of H5N1 highly pathogenic avian influenza to human health**

Authors: Juliette Blais-Savoie, BSc^1,2^, Emily Halajian, MSc^1,2^, Kuganya Nirmalarajah, BHSc^1,2^, Andra Banete, PhD^1^, Juan C. Corredor, PhD^1^, Jonathon D. Kotwa, PhD^1^, Yaejin Lee, BSc^1,2^, Sugandha Raj, PhD^3^, Shayan Sharif, PhD DVM^3^, Nicole Mideo PhD^4^, Samira Mubareka, MD^1,2^

**Supplemental Material (e-Table 3)**

| **Manufacturer** | **Device Name** | **Subtyping (H5N1)** | **Authorization** |
| --- | --- | --- | --- |
| Arbor Vita Corporation | AVantage A/H5N1 Flu Test | Yes | K083278 ^1^ |
| Healgen Scientific, LLC | Healgen COVID-19/Flu A&B Ag Combo Rapid Test Cassette (Swab) | No | EUA240010 ^2^ |
| iHealth Labs, Inc. | iHealth COVID-19/Flu A&B Rapid Test Pro | No | EUA240005 ^3^ |
| Sekisui Diagnostics, LLC | Osom Ultra Plus Flu A&B Test Kit | No | K192719 ^4^ |
| Thermo Fisher Scientific | Xpect Flu A&B | No | K092423 ^5^ |
| Watmind USA | Speedy Swab Rapid COVID-19 + Flu A&B Antigen Self-Test | No | EUA240014 ^6^ |
| Watmind USA | Speedy Swab Rapid COVID-19 + Flu A&B Antigen Test | No | EUA230037 ^7^ |
| Wondfo USA Co., Ltd. | WELLlife COVID-19 / Influenza A&B Test | No | EUA240004 ^8^ |
| Abbott Diagnostics Scarborough, Inc. | BinaxNOW Influenza A & B | No | K092223 ^9^ |
| Abbott Diagnostics Scarborough, Inc. | Alere BinaxNOW Influenza A & B Card 2 | No | K162642 ^10^ |
| Becton, Dickinson and Company | BD Veritor System for Rapid Detection of Flu A+B | No | K151291 ^11^ |
| Becton, Dickinson and Company | BD Veritor System for Rapid Detection of Flu A+B Laboratory kit | No | K160164 ^12^ |
| Becton, Dickinson and Company | BD Veritor System Flu A+B Assay | No | K151301 ^13^ |
| Becton, Dickinson and Company | BD Veritor System for Rapid Detection of Flu A+B CLIA waived kit | No | K180438 ^14^ |
| Becton, Dickinson and Company (BD) | BD Veritor System for Rapid Detection of SARS-CoV-2 & Flu A+B | No | EUA203152 ^15^ |
| CorDx, Inc. | CorDx Tyfast Flu A/B & COVID-19 Multiplex Rapid Test | No | EUA230055 ^16^ |
| CorDx, Inc. | CorDx TyFast Flu A/B & COVID-19 At Home Multiplex Rapid Test* | No | EUA240006 ^17^ |
| Diagnostic Products Corp. | PathoDX Respiratory Virus Panel, Model PKRP1 | No | K983336 ^18^ |
| Hamamatsu Photonics Electron Tube Division | Acucy Influenza A&B Test with the Acucy System | No | [K182001](https://www.accessdata.fda.gov/scripts/cdrh/cfdocs/cfPMN/pmn.cfm?ID=K182001)^19^ |
| iHealth Labs, Inc. | iHealth COVID-19/Flu A&B Rapid Test* | No | EUA230053 ^20^ |
| OSANG LLC | OHC COVID-19/Flu Antigen Test Pro | No | EUA230042 ^21^ |
| OSANG LLC | QuickFinder COVID-19/Flu Antigen Self Test* | No | EUA240007 ^22^ |
| Princeton BioMeditech Corp. | Status COVID-19/Flu A&B | No | EUA210015 ^23^ |
| Princeton BioMeditech Corp. | ViraDx SARS-CoV-2/Flu A+B Rapid Antigen Test | No | EUA220131 ^24^ |
| Princeton BioMeditech Corp. | Status COVID-19 Antigen Rapid Test for Home Use* | No | EUA220119 ^25^ |
| Princeton BioMeditech Corporation | BioSign Flu A+B | No | K133474 ^26^ |
| Quidel Corp. | QuickVue Influenza A+B Test | No | K991633 ^27^ |
| Quidel Corp. | Sofia Analyzer, Sofia Influenza A+B FIA | No | K112177 ^28^ |
| Quidel Corporation | Sofia 2 Flu + SARS Antigen FIA | No | EUA202751 ^29^ |
| SA Scientific, Ltd. | SAS FluAlert A&B Test | No | K080380 ^30^ |
| SEKISUI Diagnostics, LLC | OSOM Flu SARS-CoV-2 Combo Test | No | EUA230045 ^31^ |
| SEKISUI Diagnostics, LLC | OSOM Flu SARS-CoV-2 Combo Home Test* | No | EUA240002 ^32^ |
| Wondfo USA Co., Ltd. | WELLlife COVID-19 / Influenza A&B Home Test* | No | EUA240004 ^8^ |

e-Table 3 – Rapid diagnostic tests. Tests marked with (*) have FDA 510(k) clearance, were granted de novo request, or are authorized for emergency use (EUA) for home use. Tests marked with (**) have FDA 510(k) clearance, or granted de novo request, or are authorized for emergency use (EUA) for use with home collected specimens. Note that one FDA 510(k) clearance, granted de novo request, or emergency use authorization (EUA) is linked for each device name. There may be additional FDA 510(k) clearances, granted de novo requests, or EUAs due to changes made over time while retaining the same device name.

References

1. FDA. 501(k) Premarket Notification K083278 [Internet]. 2009 [cited 2025 Jul 18];Available from: https://www.accessdata.fda.gov/cdrh_docs/pdf8/K083278.pdf

2. FDA. Healgen COVID-19/Flu A&B Ag Combo Rapid Test Cassette (Swab) Emergency Use Authorization (EUA240010) [Internet]. 2024 [cited 2025 Jul 18];Available from: https://www.fda.gov/media/179346/download?attachment

3. FDA. iHealth COVID-19/Flu A&B Rapid Test Pro Emergency Use Authorization (EUA240005) [Internet]. 2024 [cited 2025 Jul 18];Available from: https://www.fda.gov/media/179275/download?attachment

4. FDA. 510(k) Premarket Notification K192719 [Internet]. 2020 [cited 2025 Aug 27];Available from: https://www.accessdata.fda.gov/scripts/cdrh/cfdocs/cfPMN/pmn.cfm?ID=K192719

5. FDA. 510(k) Premarket Notification K092423 [Internet]. 2009 [cited 2025 Aug 27];Available from: https://www.accessdata.fda.gov/scripts/cdrh/cfdocs/cfPMN/pmn.cfm?ID=K092423

6. FDA. Speedy Swab Rapid COVID-19 + Flu A&B Antigen Self-Test Emergency Use Authorization (EUA240014) [Internet]. 2024 [cited 2025 Jul 18];Available from: https://www.fda.gov/media/179353/download?attachment

7. FDA. Speedy Swab Rapid COVID-19 + Flu A&B Antigen Test Emergency Use Authorization (EUA230037) [Internet]. 2024 [cited 2025 Jul 18];Available from: https://www.fda.gov/media/178954/download?attachment

8. FDA. WELLlife COVID-19 / Influenza A&B Test Emergency Use Authorization (EUA240004) [Internet]. 2024 [cited 2025 Jul 18];Available from: https://www.fda.gov/media/177982/download?attachment

9. FDA. 510(k) Premarket Notification K092223 [Internet]. 2009 [cited 2025 Aug 27];Available from: https://www.accessdata.fda.gov/scripts/cdrh/cfdocs/cfPMN/pmn.cfm?ID=K092223

10. FDA. 510(k) Premarket Notification K162642 [Internet]. 2017 [cited 2025 Aug 27];Available from: https://www.accessdata.fda.gov/scripts/cdrh/cfdocs/cfPMN/pmn.cfm?ID=K162642

11. FDA. 510(k) Premarket Notification K151291 [Internet]. 2015 [cited 2025 Aug 27];Available from: https://www.accessdata.fda.gov/scripts/cdrh/cfdocs/cfPMN/pmn.cfm?ID=K151291

12. FDA. 510(k) Premarket Notification K160164 [Internet]. 2016 [cited 2025 Aug 27];Available from: https://www.accessdata.fda.gov/scripts/cdrh/cfdocs/cfPMN/pmn.cfm?ID=K160164

13. FDA. 510(k) Premarket Notification K151301 [Internet]. 2015 [cited 2025 Aug 27];Available from: https://www.accessdata.fda.gov/scripts/cdrh/cfdocs/cfPMN/pmn.cfm?ID=K151301

14. FDA. 510(k) Premarket Notification K180438 [Internet]. 2018 [cited 2025 Aug 27];Available from: https://www.accessdata.fda.gov/scripts/cdrh/cfdocs/cfPMN/pmn.cfm?ID=K180438

15. FDA. BD Veritor System for Rapid Detection of SARS-CoV-2 & Flu A+B Emergency Use Authorization (EUA203152) [Internet]. 2021 [cited 2025 Jul 18];Available from: https://www.fda.gov/media/147013/download?attachment

16. FDA. CorDx Tyfast Flu A/B & COVID-19 Multiplex Rapid Test Emergency Use Authorization (EUA230055) [Internet]. 2024 [cited 2025 Jul 18];Available from: https://www.fda.gov/media/177298/download?attachment

17. FDA. CorDx TyFast Flu A/B & COVID-19 At Home Multiplex Rapid Test Emergency Use Authorization (EUA240006) [Internet]. 2025 [cited 2025 Jun 18];Available from: https://www.fda.gov/media/177666/download?attachment

18. FDA. 510(k) Premarket Notification [Internet]. [cited 2025 Aug 27];Available from: https://www.accessdata.fda.gov/scripts/cdrh/cfdocs/cfPMN/pmn.cfm?ID=K983336

19. FDA. 510(k) Premarket Notification [Internet]. [cited 2025 Aug 27];Available from: https://www.accessdata.fda.gov/scripts/cdrh/cfdocs/cfPMN/pmn.cfm?ID=K182001

20. FDA. iHealth COVID-19/Flu A&B Rapid Test Emergency Use Authorization (EUA230053) [Internet]. 2025 [cited 2025 Jun 18];Available from: https://www.fda.gov/media/178551/download?attachment

21. FDA. OHC COVID-19/Flu Antigen Test Pro Emergency Use Authorization (EUA230042) [Internet]. 2025 [cited 2025 Jul 18];Available from: https://www.fda.gov/media/177295/download?attachment

22. FDA. QuickFinder COVID-19/Flu Antigen Self Test Emergency Use Authorization (EUA240007) [Internet]. 2024 [cited 2025 Jul 18];Available from: https://www.fda.gov/media/177575/download?attachment

23. FDA. Status COVID-19/Flu A&B Emergency Use Authorization (EUA210015) [Internet]. 2021 [cited 2025 Jul 18];Available from: https://www.fda.gov/media/145694/download?attachment

24. FDA. ViraDx SARS-CoV-2/Flu A+B Rapid Antigen Test Emergency Use Authorization (EUA220131) [Internet]. 2023 [cited 2025 Jul 18];Available from: https://www.fda.gov/media/171962/download?attachment

25. FDA. Status COVID-19 Antigen Rapid Test for Home Use Emergency Use Authorization (EUA220119) [Internet]. 2023 [cited 2025 Jul 18];Available from: https://www.fda.gov/media/167677/download?attachment

26. FDA. 510(k) Premarket Notification K133474 [Internet]. 2023 [cited 2025 Aug 27];Available from: https://www.accessdata.fda.gov/scripts/cdrh/cfdocs/cfPMN/pmn.cfm?ID=K133474

27. FDA. 510(k) Premarket Notification K991633 [Internet]. 1999 [cited 2025 Aug 27];Available from: https://www.accessdata.fda.gov/scripts/cdrh/cfdocs/cfPMN/pmn.cfm?ID=K991633

28. FDA. 510(k) Premarket Notification K112177 [Internet]. 2011 [cited 2025 Aug 27];Available from: https://www.accessdata.fda.gov/scripts/cdrh/cfdocs/cfPMN/pmn.cfm?ID=K112177

29. FDA. Sofia 2 Flu + SARS Antigen FIA Emergency Use Authorization [Internet]. 2020 [cited 2025 Jul 18];Available from: https://www.fda.gov/media/142701/download?attachment

30. FDA. 510(k) Premarket Notification K080380 [Internet]. 2009 [cited 2025 Aug 28];Available from: https://www.accessdata.fda.gov/scripts/cdrh/cfdocs/cfPMN/pmn.cfm?ID=K080380

31. FDA. OSOM Flu SARS-CoV-2 Combo Test Emergency Use Authorization (EUA230045) [Internet]. 2024 [cited 2025 Jul 18];Available from: https://www.fda.gov/media/176716/download?attachment

32. FDA. OSOM Flu SARS-CoV-2 Combo Home Test Emergency Use Authorization (EUA240002) [Internet]. 2024 [cited 2025 Jul 18];Available from: https://www.fda.gov/media/176728/download?attachment
